# Enhancing Interpretable, Transparent, and Unobtrusive Detection of Acute Marijuana Intoxication in Natural Environments: Harnessing Smart Devices and Explainable AI to Empower Just-In-Time Adaptive Interventions: Longitudinal Observational Study

**DOI:** 10.2196/52270

**Published:** 2025-01-02

**Authors:** Sang Won Bae, Tammy Chung, Tongze Zhang, Anind K Dey, Rahul Islam

**Affiliations:** 1 Human-Computer Interaction and Human-Centered AI Systems Lab AI for Healthcare Lab, Charles V. Schaefer, Jr. School of Engineering and Science Stevens Institute of Technology Hoboken, NJ United States; 2 Institute for Health Healthcare Policy and Aging Research Rutgers University Newark, NJ United States; 3 Information School University of Washington Seattle, WA United States

**Keywords:** digital phenotyping, smart devices, intoxication, smartphone-based sensors, wearables, mHealth, marijuana, cannabis, data collection, passive sensing, Fitbit, machine learning, eXtreme Gradient Boosting Machine classifier, XGBoost, algorithmic decision-making process, explainable artificial intelligence, XAI, artificial intelligence, JITAI, decision support, just-in-time adaptive interventions, experience sampling

## Abstract

**Background:**

Acute marijuana intoxication can impair motor skills and cognitive functions such as attention and information processing. However, traditional tests, like blood, urine, and saliva, fail to accurately detect acute marijuana intoxication in real time.

**Objective:**

This study aims to explore whether integrating smartphone-based sensors with readily accessible wearable activity trackers, like Fitbit, can enhance the detection of acute marijuana intoxication in naturalistic settings. No previous research has investigated the effectiveness of passive sensing technologies for enhancing algorithm accuracy or enhancing the interpretability of digital phenotyping through explainable artificial intelligence in real-life scenarios. This approach aims to provide insights into how individuals interact with digital devices during algorithmic decision-making, particularly for detecting moderate to intensive marijuana intoxication in real-world contexts.

**Methods:**

Sensor data from smartphones and Fitbits, along with self-reported marijuana use, were collected from 33 young adults over a 30-day period using the experience sampling method. Participants rated their level of intoxication on a scale from 1 to 10 within 15 minutes of consuming marijuana and during 3 daily semirandom prompts. The ratings were categorized as not intoxicated (0), low (1-3), and moderate to intense intoxication (4-10). The study analyzed the performance of models using mobile phone data only, Fitbit data only, and a combination of both (MobiFit) in detecting acute marijuana intoxication.

**Results:**

The eXtreme Gradient Boosting Machine classifier showed that the MobiFit model, which combines mobile phone and wearable device data, achieved 99% accuracy (area under the curve=0.99; *F*_1_-score=0.85) in detecting acute marijuana intoxication in natural environments. The *F*_1_-score indicated significant improvements in sensitivity and specificity for the combined MobiFit model compared to using mobile or Fitbit data alone. Explainable artificial intelligence revealed that moderate to intense self-reported marijuana intoxication was associated with specific smartphone and Fitbit metrics, including elevated minimum heart rate, reduced macromovement, and increased noise energy around participants.

**Conclusions:**

This study demonstrates the potential of using smartphone sensors and wearable devices for interpretable, transparent, and unobtrusive monitoring of acute marijuana intoxication in daily life. Advanced algorithmic decision-making provides valuable insight into behavioral, physiological, and environmental factors that could support timely interventions to reduce marijuana-related harm. Future real-world applications of these algorithms should be evaluated in collaboration with clinical experts to enhance their practicality and effectiveness.

## Introduction

### Background

Acute effects of marijuana use impair motor skills and cognitive functions, such as attention and information processing [1-3], leading to adverse outcomes like poor academic and work performance, as well as an increased risk of motor vehicle crashes and fatal collisions [2,4]. Delta-9 tetrahydrocannabinol (THC), the principal psychoactive constituent of marijuana, binds to brain receptors, inducing a feeling of “euphoria” or being “high” [5]. Given the risks associated with THC-induced impairment, there is a critical need to detect episodes of marijuana intoxication in real time in the natural environment.

Several studies have explored the use of phone sensors or wearable devices to detect acute marijuana consumption. For example, a laboratory study with 10 participants used smartphone sensors (accelerometer, gyroscope) to detect acute marijuana use (3% or 7% THC vs placebo) and found that gait analysis with a support vector machine model achieved 92% accuracy (*F*_1_-score=0.93) [6]. Another study (n=1) developed an electrochemical biosensor ring that detected salivary THC (minimum of 0.5 μM) and blood alcohol levels (minimum of 0.2 mM) within three minutes [7]. However, these studies were conducted in controlled environments, highlighting the need for research on using smartphone and wearable sensors to detect acute marijuana use in nonlaboratory, natural settings.

Detecting marijuana use in daily life could enable Just-In-Time interventions to reduce harm, such as avoiding driving while intoxicated [8]. However, challenges exist in detecting acute marijuana-related intoxication [9]. THC could be detected in an individual’s blood or urine for several days after consumption depending on factors such as recency, frequency, and chronicity of use [10]. Thus, a person who tests positive for THC might not be intoxicated or impaired at the time of testing [10]. Existing testing methods (eg, blood, urine, saliva, and breath) are not suitable for real-time detection, as THC can remain detectable in the body for days after consumption, which does not necessarily indicate current impairment [10].

To address these limitations, our recent study [11] used passive sensing via smartphones, coupled with self-reported intoxication, to detect marijuana use with 90% accuracy, using sensor-derived data from mobile phones alongside temporal variables, including time of day and day of week. Building on these findings [11], this study explores the use of wearable devices (eg, Fitbit) to enhance detection capabilities by incorporating physiological indicators, thereby improving the accuracy and immediacy of identifying marijuana effects in natural environments.

Wearable device–reported heart rate (HR) was examined as a potential physiological indicator of acute marijuana intoxication, based on laboratory studies, showing a dose-dependent increase in resting HR shortly after smoking or vaping marijuana [12-14]. Specifically, laboratory research reports that within 2-3 minutes of smoking marijuana, there is an acute increase (20%-60% dose-dependent) in resting HR [13], which might represent a “physiological signal” of the onset of a marijuana smoking episode. HR peaks 10-15 minutes after reaching maximum THC levels, followed by a rapid decline [12-14]. While tolerance to this effect may develop (eg, from a mean increase of 44.6 to 6.6 beats per minute (bpm) after 18-20 days of use) with chronic use, [12-14]. The acute HR increases have been validated in laboratory settings but have remained unexplored in real-world contexts. This study examines using off-the-shelf wearable devices, such as Fitbit, to detect acute HR increases as a physiological signal potentially correlated with self-reported marijuana intoxication.

### Research Objectives and Contributions

While laboratory studies have established the link between HR changes and marijuana intoxication [12-14], its applicability in real-world scenarios is unexplored. To address this gap, we propose that combining wearable device data with smartphone sensors could improve algorithms for detecting marijuana intoxication in real-life settings. To enhance the interpretability of our algorithms and provide insights for just-in-time adaptive interventions, we incorporated explainable artificial intelligence (XAI) into our machine-learning pipeline. XAI helps clarify the role of digital biomarkers associated with self-reported marijuana intoxication in natural environments.

This study aims to determine whether data from smartphones (eg, accelerometer and GPS) and wearable devices (eg, Fitbit) can detect self-reported marijuana intoxication (“feeling high”) in the natural environment, a topic not previously investigated. Two hypotheses drive this research: (1) the novel MobiFit model, which combines smartphones and Fitbit data will outperform models that use only one data source in detecting self-reported intoxication; (2) HR and daily behavioral data (eg, step count) from Fitbit are important features for detecting self-reported marijuana intoxication. If either hypothesis is validated, it indicates the value of integrating wearable device data into daily life monitoring.

This study evaluates the performance of sensor-based models using (1) only smartphone sensors, (2) only Fitbit data, and (3) the combined MobiFit model. We also used XAI to enhance understanding of key digital features from both smartphone sensors and Fitbit data associated with self-reported marijuana intoxication. Identifying smartphone-based sensors and Fitbit features that accurately detect self-reported marijuana intoxication in natural environments could ultimately trigger just-in-time interventions.

This study presents a comprehensive approach toward using mobile and wearable technology for detecting self-reported acute marijuana intoxication in real-life settings, emphasizing interpretability and transparency through XAI. This study demonstrates the potential of integrating smart devices with advanced analytical techniques to improve detection accuracy and support timely interventions based on detected intoxication levels.

## Methods

### Recruitment and Participants

A total of 57 participants aged 18-24 years were recruited through flyers, advertisements, and local communities. Eligibility criteria were (1) using marijuana at least twice a week, (2) owning a personal mobile phone, (3) not currently seeking treatment for substance abuse, (4) no self-reported history of psychosis, and (5) not taking any medication or using any medical device (eg, pacemaker) that could affect HR. Of the 57 participants, 24 participants were excluded from the analysis due to missing data (eg, no HR data and no mobile sensor data).

The final analysis focused on 33 participants aged 18-24 years, with an average age of 19.64 (SD 1.77) years. Among these, 23 participants identified as White, 4 participants as Black, and 6 participants as other race or ethnicity. The average age of first marijuana use was 16.48 (SD 1.84, range 13-22) years, and the average age of regular marijuana use was 17.03 (SD 1.72) years. In this subset, 24% (n=8) reported daily marijuana use, 9% (n=3) reported using it 5-6 times per week, and 67% (n=22) reported using it 2-4 times per week. Notably, 97% (n=32) of participants primarily used iOS smartphones, with only 3% (n=1) using Android devices.

### Ethical Considerations

This naturalistic, observational follow-along study was approved by the university’s institutional review board (Stevens 2020-008 [23-COAS3], Rutgers Pro2019002365). In line with similar Institutional Review Board–approved observational studies [15], all participants were informed about local medical and mental health resources. The study obtained a National Institutes of Health Certificate of Confidentiality. Written consent was obtained from participants, who were informed about privacy protections and the voluntary nature of their participation [16]. The research staff explained the types of data to be collected, the duration of data collection, and the purpose of the study.

### Study Design

Participants completed a baseline laboratory assessment including interviews, questionnaires, and cognitive testing. They downloaded study apps from the App Store or Google Play Store to their smartphones. Research staff trained participants on how to use the apps and the study provided Fitbit Charge 2 for data collection. The AWARE mobile app [17] delivered experience sampling method (ESM) questions on marijuana use. Participants wore the Fitbit Charge 2 wristband to collect data on HR, physical activity (eg, step count), and sleep (eg, time, duration, and quality; see Table S2 in Multimedia Appendix 1 for Fitbit variables). The study collected continuous sensor data from smartphones and Fitbit devices, along with self-reported data on marijuana intoxication, for up to 30 days. A 30-day period was chosen to ensure sufficient data, given the study’s inclusion criteria of frequent marijuana use. At the end of the study, participants completed a debriefing interview about their experience.

Participants were compensated for their time and effort, receiving US $75 for completing the baseline assessment, and US $25 for the debriefing interview. They earned US $10 for each day on which they completed more than 75% of data collection (eg, Fitbit and ESM).

### Mobile Sensing Framework and Applications for Data Collection

#### AWARE App

AWARE is a mobile sensing framework [17] that passively and continuously collects data from smartphone sensors. This data can be used to infer human behavior patterns using various sensors: location (eg, distance traveled and circadian rhythm), physical movements (eg, acceleration and activity), device usage (eg, unlock, charge, keypress, and app usage), social patterns (eg, communication and conversations), and environmental context (eg, Wi-Fi, Bluetooth, sound or ambient noise, and light). The app, developed to track participants’ natural behaviors in real-life settings, runs in the background 24/7 and collects sensor data with associated metadata, such as time stamps and communication logs. The data is transferred to a secure MySQL database owned and operated by the research team.

#### ESM

The mobile app also captured self-reports of marijuana use by participants. Two types of surveys were used [18]. Participants manually reported marijuana use within 15 minutes of consumption, detailing the amount used, mode of consumption, and the people whom the participant consumed marijuana with. They also rated their subjective intoxication on a scale from 0 (none) to 10 (a lot) [19]. Two hours later, the app prompted participants to complete an end-session survey indicating when intoxication symptoms subsided. In addition, fixed-time surveys were delivered daily at 10 AM, 3 PM, and 8 PM to collect information on the participants’ daily lives, including time since last marijuana use, cravings, mood, and feelings (eg, relaxed, anxious, and sad), and other substance use (eg, alcohol and tobacco). Survey response windows were open for 5 hours to accommodate participants’ schedules.

#### Fitbit Charge 2

Participants were provided with Fitbit Charge 2 devices and asked to wear them as much as possible. Fitbit collected physiological data (eg, HR), activity data (eg, step count), and sleep. The study hypothesized that HR and behavioral data could signal episodes of acute marijuana intoxication. Fitbit data were retrieved from the Fitbit server at the end of the study using the Fitbit application programming interface.

### Preparing Self-Report and Fitbit Data for Analysis

An episode of self-reported subjective marijuana intoxication was defined based on the ESM item: “How high are you feeling right now?” rated from 0 to 10 (0=not high to 10=a lot) [18,19]. To include episodes in the analysis, both start and end times had to be reported to calculate duration and label the sensor data. To capture behaviors without marijuana use, 1556 reports where participants answered “no” to the question “Did you smoke marijuana since the last report?” during afternoon (n=1151) and evening (n=950) surveys were labeled as “0” for the subjective rating of marijuana intoxication.

**Figure 1:**
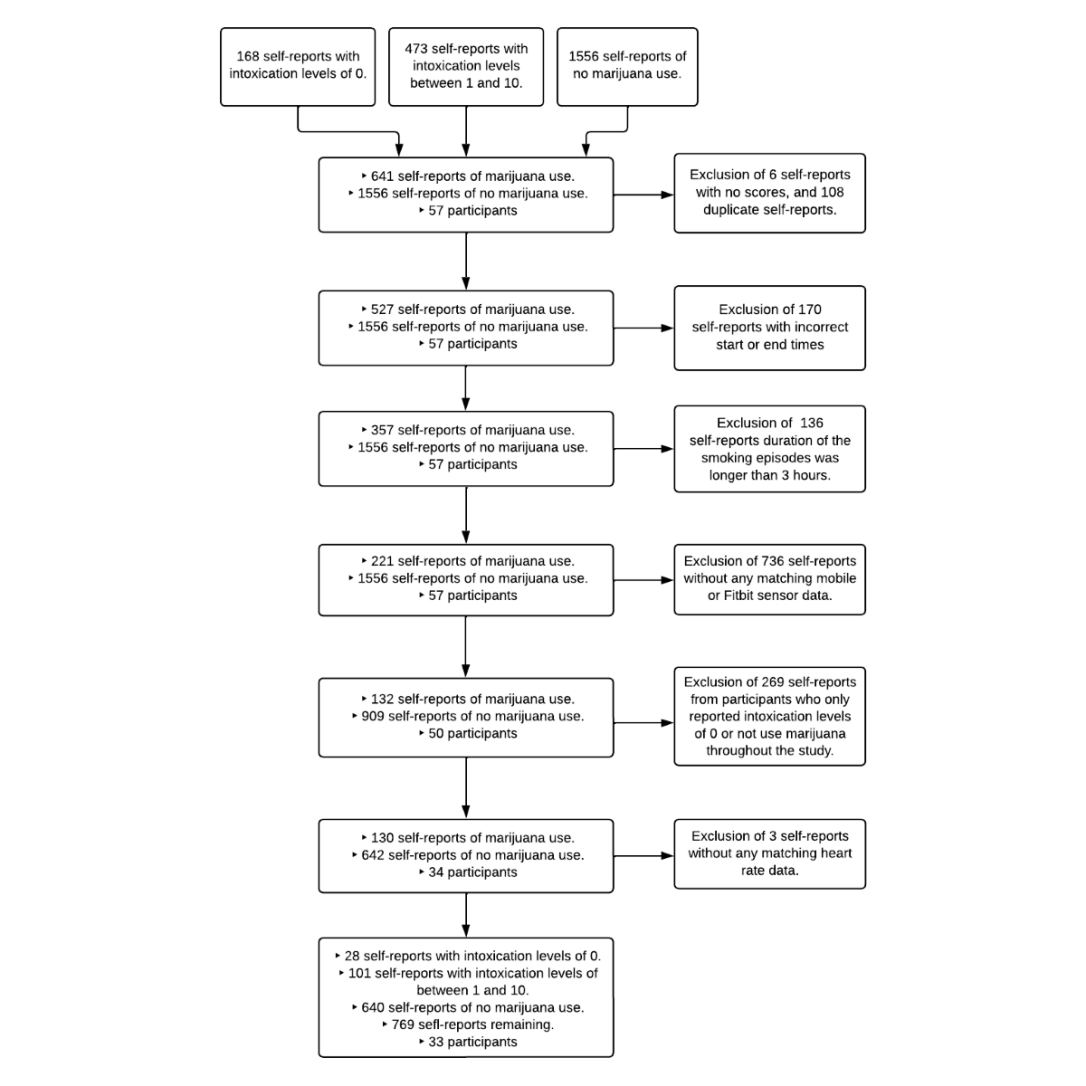
Flowchart of participants and the data included in the analyses.

From all participants, we received 641 self-reports (mean 9.86, SD 8.49; median 7, IQR 4-13) and 1556 with no marijuana use reports (Figure 1). Out of 641 reports, 168 reports had a subjective intoxication rating of 0 and 10, and 6 reports had no rating. After excluding 6 reports without ratings and 108 duplicate reports, 527 samples remained. Reports with missing start and end times, or implausible episode durations (eg, longer than 3 hours) were excluded based on laboratory research indicating that smoked or vaped marijuana effects last less than 3 hours [20]. A total of 136 self-reports were excluded for exceeding this duration, leaving 1556 reports where no marijuana use was recorded [20].

For model building, episodes without mobile sensor data (n=72) were excluded, leaving 221 marijuana self-reports. Furthermore, episodes without Fitbit sensor data (n=17) were excluded, leaving 50 participants. These participants provided 132 marijuana use self-reports and 909 “no marijuana use” reports. We analyzed reports from each participant, excluding those who only reported not using marijuana or had a rating of 0 for subjective intoxication, leaving a total of 642 with no marijuana use report or who reported 0 subjective intoxications when using marijuana and 34 people. Finally, to prevent participants from using Fitbit incorrectly, we excluded users without HR data, leaving a total of 33 people, who provided a total of 769 events: 640 “no marijuana use” reports and 129 marijuana use self-reports.

### Extracting Smartphone and Fitbit Sensor Features

Following previous studies, we extracted audio features to detect social interactions [21,22] potentially associated with marijuana use. Audio features were extracted using the conversation plug-in, which detects whether a person was engaged in a conversation. Raw audio signals are converted to amplitude using the Euclidean norm [23], which categorizes ambient levels into silence, noise, voice, and unknown [24]. We also computed device use features, such as smartphone unlock minutes and the duration of device interaction sessions. In addition to audio features, we extracted GPS features to examine movement patterns related to marijuana use [25-28]. These included the radius of gyration, time at a location cluster, total distance traveled, number of clusters within a 5-minute window, acceleration, and phone angles. Environmental features, such as the number of Bluetooth devices detected, the most frequently contacted Wi-Fi access point, and light features (eg, average [avg], and maximum [max] lux) were also extracted. For most features, we calculated the minimum (min), max, avg, median (med), and SD. Further details on smartphone features can be found in Multimedia Appendix 1.

We used a 5-minute time window for extracting sensor feature statistics, as laboratory studies show a dose-dependent acute in resting HR within 2-3 minutes of marijuana use. Using larger time intervals could include data not related to marijuana use, given the average reported marijuana session duration is 75 (SD 46.2) minutes.

Raw data for HR, sleep, and steps were extracted from Fitbit. We first obtained per-minute HR and step count data using the Fitbit application programming interface. To exclude outliers, we refined data selection to omit instances where HR was below 40 bpm, as recommended by the American Heart Association [29,30]. We extracted feature statistics such as avg, SD, min, med, and max HR within a 5-minute window to explore the relationship between HR and marijuana intoxication levels (“moderate-intensive,” “low,” and “none”). Resting HR was defined as HR data collected when the participant was sedentary (ie, no steps taken) for more than 5 minutes. To further analyze HR patterns related to marijuana intoxication, we examined the degree of peakedness (kurtosis) and asymmetry (skewness) in HR data, as these features may reveal physiological changes associated with marijuana intoxication [31]. For more details, refer to Table S2 in Multimedia Appendix 2.

### Ground Truth and Labeling Sensor Data

**Figure 2:**
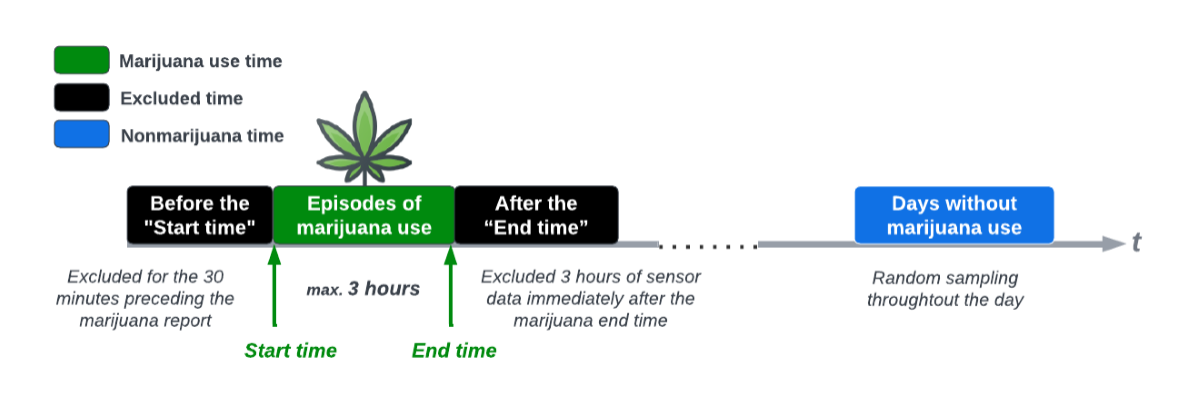
Marijuana use episodes and labeling principle.

To accurately label the collected sensor data, we defined the duration of marijuana use episodes as those equal to or less than 3 hours, based on reported start and end times. We excluded 3 hours of sensor data following the reported end time to account for the continued effects of marijuana, even when participants reported a subjective intoxication level of 0. For example, if marijuana use was reported from 6 PM to 6:30 PM, data from 6:30 PM to 9:30 PM were excluded to account for residual effects. We also excluded data from 30 minutes before the reported start time to account for potential delays in self-reporting, based on pilot study findings that delays could range from 5 to 15 minutes. To collect nonmarijuana data, we randomly sampled sensor data from days when participants did not use marijuana (ie, nonmarijuana days). These samples were labeled using morning, afternoon, and evening surveys in which participants reported “no” to the ESM item “Did you smoke marijuana since the last report?” and indicated that the last use was more than 5 hours before the ESM time stamp (Figure 2).

We aimed to capture acute intoxication versus nonuse, classifying intoxication levels into three categories: 0 as “not intoxicated,” 1-3 as “low intoxication,” and 4-10 as “moderate-intensive intoxication” (MI). In total, we labeled 32,722 sensor stream samples (5-minute windows) as “not intoxicated” (154 from self-initiated survey coded as 0 high, and 32,586 from time-based self-reports), 423 samples as “low intoxication” (ratings between 1 and 3) and 772 samples as “moderate-intensive” (ratings between 4 and 10, with 10 indicating “a lot”).

Data from smartphones and Fitbit resulted in two datasets of different sizes. To ensure consistency, we down-sampled the smartphone dataset to include only samples overlapping with Fitbit data during the same time frames. This resulted in three datasets: (1) eXtreme Gradient Boosting (XGBoost)-Mobile: mobile phone only, (2) XGBoost-Fitbit: Fitbit-only, and (3) XGBoost-MobiFit: combined mobile and Fitbit data. The rationale for choosing Machine Learning (ML) models is detailed in Multimedia Appendix 3 and model comparison with different classifiers can be found in Multimedia Appendix 4.

### ML Pipeline

#### Feature Selection

**Figure 3:**
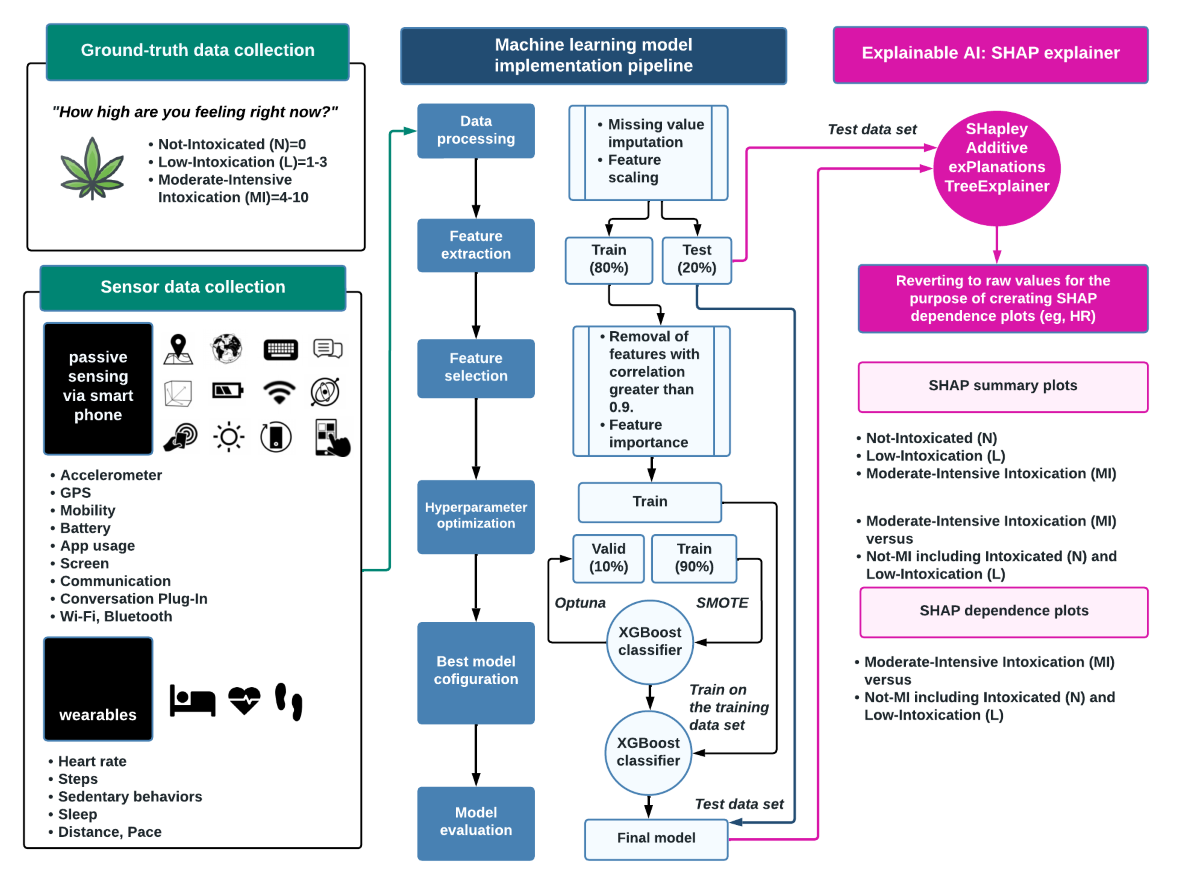
Study overview. AI: artificial intelligence; HR: heart rate; SHAP: Shapley Additive exPlanations; SMOTE: Synthetic Minority Over-Sampling Technique; XGBoost: eXtreme Gradient Boosting Machine.

We began data analysis by randomly partitioning the labeled sensor data into training (80%) and test (20% holdout) datasets. As shown in Figure 3, we first calculated Pearson correlation coefficients between features in the training dataset to identify highly covariant feature pairs (correlation coefficients >0.9) [32]. We then systematically removed one feature from each pair to reduce redundancy and improve model performance by retaining the most relevant and independent features. Next, we selected statistically significant features with a Gini coefficient importance [33] greater than 0.005. Details can be found in Multimedia Appendix 2.

#### Hyper-Parameter Tuning and Cross-Validation

As shown in Figure 3, during hyper-parameter tuning in the training dataset, we used cross-validation to randomly leave 10% of the samples out, training the model on the remaining 90% and testing on the withheld 10%. We used the Synthetic Minority Over-Sampling Technique [34] to ensure equal representation across all classes. We further optimized model performance with a Bayesian-optimization-driven method called Optuna [35] to select the best combination of hyperparameters and 10-fold cross-validation on models with Optuna-optimized hyperparameters.

For the final model evaluation, we used the reserved test data (20% unseen data, as shown in Figure 3). The model was evaluated on predictions made on the test data. Finally, as shown in Figure 3 (right column), we conducted an XAI analysis to better understand the decision-making process of our final predictive model. We generated SHapley Additive exPlanations (SHAP) on the unseen test data to ensure our findings were explainable for data the model had not seen.

### Model Evaluation Metrics

We evaluated model performance using *F*_1_-score, recall, and precision, and selecting the best model based on the *F*_1_-score [36]. Low precision indicates too many false positives (ie, detecting intoxication when there is none), here we would mistakenly intervene or notify the participant. Low recall indicates too many false negatives (ie, not detecting intoxication when it occurs), potentially leading to unsafe behaviors such as impaired driving. Therefore, while we prioritize the *F*_1_-score, we also consider precision and recall.

Given our imbalanced samples, we used the area under the curve (AUC) metric, which provides a robust evaluation across all classification thresholds and is resilient to class imbalance.

### XAI: Interpretation Approaches for Black-Box ML Models

To enhance algorithmic transparency, we used SHAP, a widely used interpretability method for ML models [37,38]. SHAP explains how specific data features influence model predictions, providing insights into the model’s decision-making process. We identified the top 30 most significant features associated with marijuana intoxication reports, including their importance scores and visual summaries calculated by SHAP (see “Key Features Contributing to Model Performance” under the Results section). XGboost was selected due to its superior performance compared to other classifiers. The use of tree SHAP in this context reduces the computation time for SHAP values from exponential to polynomial [37].

## Results

### Timing, Duration, and Rating of Subjective Marijuana Intoxication

During the 30-day period, participants averaged 14 (SD 8.59) days of active participation. A total of 129 ESM self-initiated reports of marijuana use met the criteria for inclusion in the analysis: 101 reports of subjective marijuana intoxication (feeling high rated 1-10 out of 10) and 28 reports of feeling not high (0). Events not involving marijuana use were assigned a high rating of 0.

Tables 1 and 2 show the distribution of self-reported subjective marijuana intoxication across participants. Most episodes of intoxication (n=75) lasted between 30 minutes and 3 hours, with 54 episodes lasting up to 30 minutes (Table 1). Marijuana use was most often reported between 10 PM and 11 PM (n=24). Table 2 shows the distribution of ESM responses throughout the day. The average response latency to an ESM prompt expired. Most self-initiated reports of marijuana use occurred in the evenings: 14% (n=18) between 6 PM and 9 PM, and 39% (n=50) between 9 PM and midnight. On average, young adults rated their feeling of being high at 3.63 (SD 2.72) out of 10 when using marijuana (Table 3).

**Table 1:** Distribution of the duration of self-reported marijuana use episodes (n=129) across participants.

Duration^a^ (hours)	Number of events
<0.5	54
<1	20
<1.5	23
<2.0	13
<2.5	13
<3	6

^a^Duration refers to the window of smoking episodes. From small (30 minutes) to relatively large windows (3 hours).

**Table 2:** Distribution of the start time of marijuana use episodes during the day (n=129).

Clock time (hours)	Number of events
0-1	7
1-2	8
2-3	2
3-4	0
4-5	0
5-6	0
6-7	0
7-8	1
8-9	0
9-10	5
10-11	8
11-12	2
12-13	6
13-14	6
14-15	5
15-16	4
16-17	3
17-18	4
18-19	5
19-20	6
20-21	7
21-22	10
22-23	24
23-0	16

**Table 3:** Distribution of self-reported “feeling high” during marijuana use.

High rating^a^	Number of events
0	28
1	9
2	9
3	17
4	14
5	14
6	17
7	10
8	7
9	4
10	0

^a^0-10 scale representing an intensity of feeling high, 10=a lot from the self-initiated reports of marijuana use. In our study, a value of 0 for the high report is labeled as “no-intoxication.”

### Model Comparison: Mobile Only, Fitbit Only, and Mobile and Fitbit Integration

The first part of our analysis aimed to determine whether smartphone sensor features alone could be used for real-time detection of subjective marijuana intoxication and whether adding Fitbit data would improve model performance, justifying the added complexity of Fitbit data collection. We compared three ML models using the XGBoost classifier: (1) smartphone sensors only (XGBoost-Mobile), (2) Fitbit features only (XGBoost-Fitbit), and (3) a combined model using smartphone and Fitbit features (XGBoost-MobiFit).

**Figure 4:**
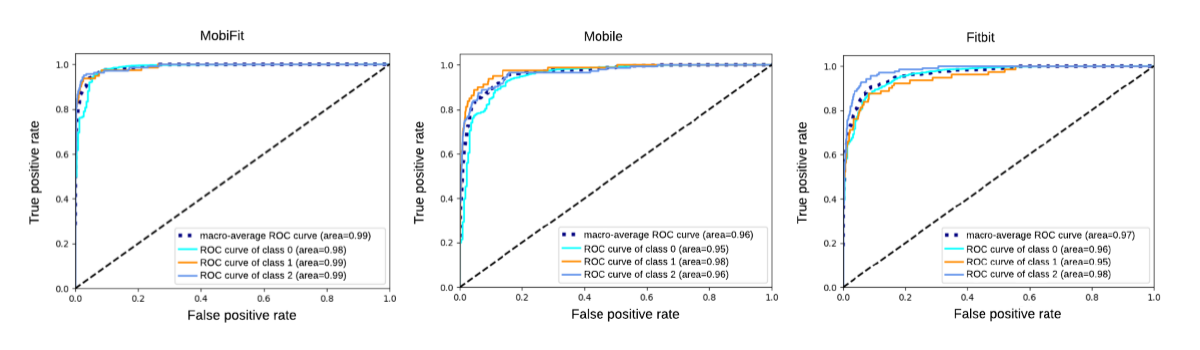
Model comparison to detect acute marijuana intoxication “low-intoxicated” (rating=1-3) versus “moderate-intensive intoxicated” (rating= 4-10) versus “not-intoxicated” (rating=0). XGBoost-MobiFit: phone sensors and Fitbit (AUC=0.99; accuracy=0.99; left), XGBoost-Mobile: smartphone-based sensors (samples overlapping with Fitbit; AUC=0.96; accuracy=0.97; middle) and XGBoost-Fitbit: Fitbit only (AUC=0.97; accuracy=0.98; right). AUC: area under the curve; ROC: receiver-operating characteristic curve; XGBoost: eXtreme gradient boosting.

Among the 3 models tested, the XGBoost-MobiFit model, which integrates smartphone and Fitbit data, had the best performance, achieving 99% accuracy, 92% precision, 79% recall, 85% *F*_1_-score, and 99% AUC on the test dataset (Figure 4 and Table 4). These metrics indicate the XGBoost-MobiFit model’s superior ability to accurately identify MI compared to low-intoxication and not-intoxicated states. While the XGBoost-Fitbit performed reasonably well, it did not match the performance of the XGBoost-MobiFit model in detecting marijuana intoxication. XGBoost-Fitbit achieved accuracy of 98%, 79% precision, 70% recall, 74% *F*_1_-score, and 97% AUC. These results suggest that using only Fitbit data may not be as effective as combining it with smartphone sensor data for detecting subjective marijuana intoxication. Based on these findings, the added burden of wearing and charging the Fitbit device seems justified in future deployments. The combined model (XGBoost-MobiFit) demonstrated improved performance in detecting subjective marijuana intoxication compared to using smartphone or Fitbit data alone.

Combining Fitbit data with mobile data resulted in a significant improvement over the Fitbit-only model. The mobile-only model achieved an AUC of 96%, an *F*_1_-score of 72%, a recall of 75%, and a precision of 70%. These results indicate that including Fitbit data adds value beyond what can be achieved with smartphone-based sensor data alone, as evidenced by a 13% improvement in *F*_1_-score.

In summary, three key findings emerged: the XGBoost-Mobile model had the lowest performance (*F*_1_-score=0.72, recall=0.75, precision=0.70); the XGBoost-Fitbit model (*F*_1_-score=0.74, recall=0.70, precision=0.79) generally performed lower than the combined model; and the XGBoost-MobiFit model was the best performer with an *F*_1_-score of 0.85, recall of 0.79, and precision of 0.92. As highlighted earlier, high precision and recall are critical so we focused on the *F*_1_-score to identify the best-performing model. The model comparison with different classifiers is provided in Multimedia Appendix 4.

**Table 4:** Comparison of three XGBoost models using features selected in detecting moderate-intensive marijuana intoxication, low-intoxication, and not-intoxicated classes on the test dataset.

Machine learning model	AUC^a^	*F*_1_-score	Recall	Precision	Accuracy
XGBoost-MobiFit	0.99	0.85	0.79	0.92	0.99
XGBoost-Mobile	0.96	0.72	0.75	0.70	0.97
XGBoost-Fitbit	0.97	0.74	0.70	0.79	0.98

^a^AUC: area under the curve.

### Understanding Model Performance in Detecting the Risk State of “Moderate and Intensive Marijuana Intoxication”

For predicting the MI class alone, the MobiFit model outperformed the mobile and Fitbit-only models, exhibiting a substantial improvement in the *F*_1_-score of 20% and 18%, respectively (Table 5). This improvement in *F*_1_-score highlights the benefits of integrating data from both devices: enhanced precision and recall for the MI class compared to the not-intoxicated (N) and low-intoxicated (L) classes (Table 6). The XGBoost-Mobile model exhibited a notably high false negative rate for instances labeled as “not-intoxicated,” often misclassifying them as “moderate-intensive intoxicated.” However, it showed better accuracy in distinguishing “low-intoxicated” instances. In contrast, the XGBoost MobiFit model demonstrated a higher true positive rate compared to the other models, accurately identifying 76% of MI samples among the total samples belonging to that class. While the XGBoost-Mobile and Fitbit models achieved recall rates of 61% and 63% in predicting MI, they incorrectly predicted 56 and 53 out of 143 actual MI samples as other classes. In comparison, the best-performing MobiFit model achieved 108 true positives out of the 143 actual MI samples. The higher precision of the MobiFit model further supports its superior performance, though there remains room for improvement as it missed 35 samples, as shown in Table 6.

**Table 5:** Performance comparison of three XGBoost^a^ models in detecting the subjective sense of moderate-intensive marijuana intoxication class.

ML^b^ model	MI^c^ precision	MI recall	MI *F*_1_-score	MI AUC^d^
XGBoost-MobiFit	0.89	0.76	0.82	0.99
XGBoost-Mobile	0.64	0.61	0.62	0.96
XGBoost-Fitbit	0.65	0.63	0.64	0.98

^a^XGBoost: eXtreme Gradient Boosting.

^b^ML: machine learning

^c^MI: moderate-intensive intoxication.

^d^AUC: area under the curve.

**Table 6:** Confusion matrix for XGBoost-MobiFit, XGBoost-Mobile, and XGBoost-Fitbit model for 3 classes.

				Predicted				
				N^a^		L^b^		MI^c^
**XGBoost^d^** **-MobiFit**								
	**Actual**							
		N	6541		7		13	
		L	29		50		1	
		MI	35		0		108	
**XGBoost-Mobile**								
	**Actual**							
		N	6452		59		50	
		L	28		52		0	
		MI	56		0		87	
**XGBoost-Fitbit**								
	**Actual**							
		N	6499		14		48	
		L	41		39		0	
		MI	52		1		90	

^a^N: not-intoxicated.

^b^L: low-intoxication.

^c^MI: moderate-intensive intoxication.

^d^XGBoost: eXtreme Gradient Boosting.

### Key Features Contributing to Model Performance

#### Overview

To explore the algorithms’ performance in predicting the MI class, we used SHAP summary visualizations [37,38] to identify patterns of acute marijuana intoxication. We determined the key features contributing significantly to the model’s predictions based on mean absolute SHAP values across all instances, with a focus on the MI class.

**Figure 5:**
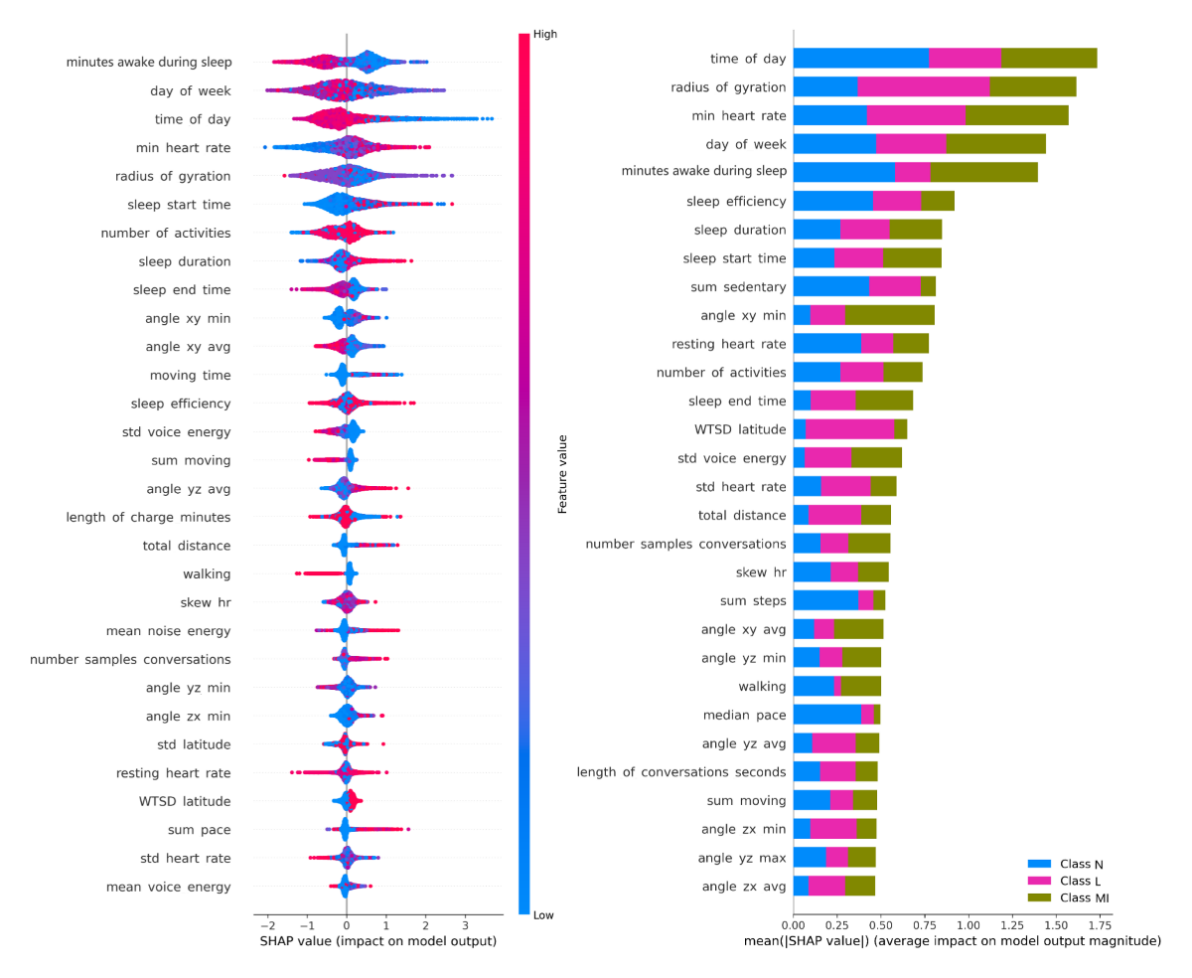
Explanations generated by SHAP summary plot. Impact of features on best performing XGBoost-MobiFit model (left) and binary model output identifying moderate-intensive intoxication (MI; SHAP>0) from nonmoderate-intensive intoxication (N and L) classes (SHAP<0; right). HR: heart rate. SHAP: SHapley Additive exPlanations; WTSD: weighted stationary latitude and longitude standard deviation; XGBoost: eXtreme Gradient Boosting.

**Figure 6:**
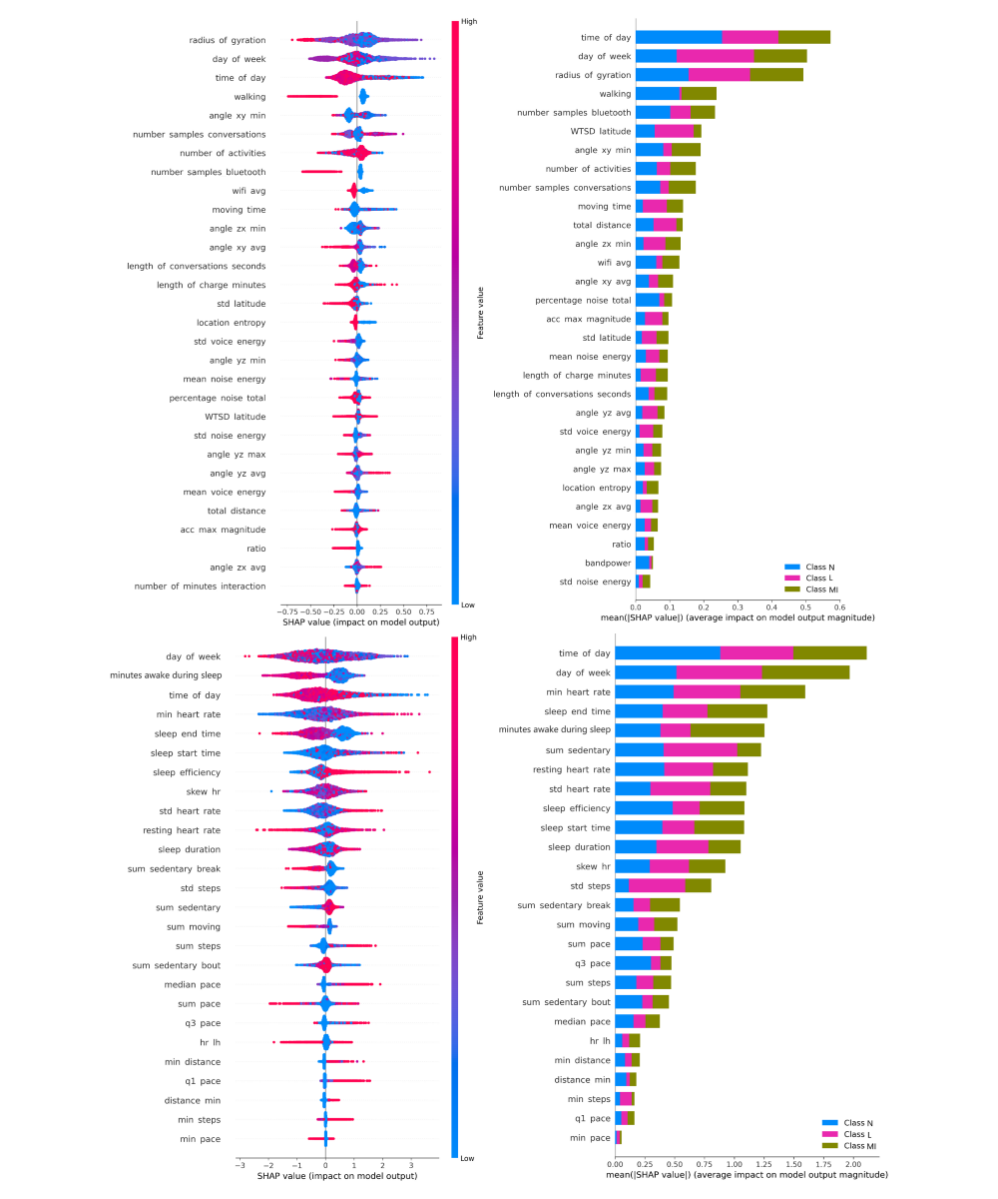
Explanations generated by SHAP summary plot. Impact of features on XGBoost-Mobile model (top left) and binary model output identifying MI (SHAP>0) from nonmoderate-intensive intoxication (N and L) classes (SHAP<0; top right), impact of features on XGBoost-Fitbit model (bottom left) and binary model output identifying MI (SHAP>0) from nonmoderate-intensive intoxication (N and L) classes (SHAP<0; bottom right). MI: moderate-intensive intoxication; SHAP: SHapley Additive exPlanations; WTSD: weighted stationary latitude and longitude standard deviation; XGBoost: eXtreme Gradient Boosting.

Figures 5 and 6 present the SHAP visualizations. In Figure 5, the length of each bar on the left indicates the feature’s contribution to the model, with longer bars signifying a stronger influence on the outcome. The SHAP summary plots on the right of Figure 5 illustrate how features influence the MI prediction class, with the strongest influence at the top. The color shading indicates the direction of the feature’s effect, with blue for low values, purple for median values, and red for high values. Plots extending to the left indicate a negative contribution to the prediction, while those extending to the right positively contribute to MI predictions.

#### Impact of Average Key Features on Model Output Magnitude

The top five influential features in detecting the three classifications (Figure 5, left) and affecting the MI outputs (Figure 5, right) included time of day, radius of gyration, minimum HR, day of the week, and minutes awake during sleep. Among physical activities and physiological signals, a diverse range of features extracted from various sensors, including those beyond time-based attributes from both mobile and Fitbit combined sensors, was chosen as the top 30 crucial elements for distinguishing between not-intoxicated (N), low-intoxication (L), and MI. The SHAP value, signifying the average impact magnitude on the model’s output, played a pivotal role in this determination (Figure 5, left).

#### Impact of Unique Key Features on Mobile and Fitbit Model Outputs

Similar to the best-performing MobiFit model, the Mobile model (Figure 6) highlighted key features with overlapping impacts on the model’s outcomes. The only exception was in specific movement and environmental context features, as shown in the top left and right graphs of Figure 6. However, the Fitbit model showed a more significant impact on HR features, with all four HR features ranking within the top 10 for all three classes (shown in the bottom-left graph in Figure 6), and for the MI classes compared to the non-MI classes (bottom-right graph in Figure 6).

### Key Features Explaining MI

#### Overview

To specifically examine the influence of key features on the “risk” state of MI, we present comprehensive details for each key feature within the model.

**Figure 7:**
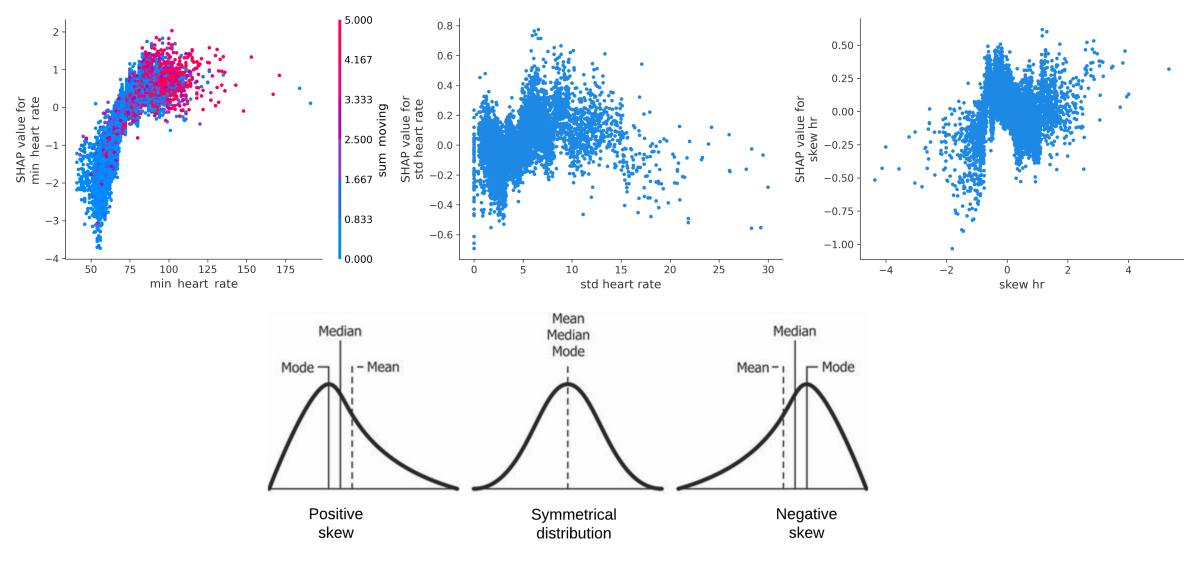
Interaction effects of total minutes spent moving on minimum HR values (top left), SD (top middle), and skewness (top right) of HR, and an explanation of skewness [39] (bottom). HR: heart rate; SHAP: SHapley Additive exPlanations.

A partial dependence plot (PDP) in Figure 7 illustrates the overall relationship between a feature and the outcome. The vertical axis represents SHAP values, signifying the effect of the chosen feature on predictions, while the horizontal axis represents actual feature values across instances. Each point represents an instance’s feature value and its corresponding SHAP value. An upward PDP slope indicates a positive impact of the feature on MI prediction, while a downward slope indicates a negative impact. The surface on the PDP plot (eg, min HR and sum of moving minutes in Figure 7, top left) shows the combined impact of the two features on MI predictions, with greater values corresponding to increased prediction values.

In the following section, we introduce the key features contributing to MI, including elevated and fluctuating HR, reduced large-scale movement patterns, increased ambient noise and voice energy, and extended sleep patterns.

#### Elevated and Fluctuating HRs

We investigated the impact of recent physical activity (measured as the sum of minutes spent moving based on Fitbit data) on HR in relation to self-reported marijuana intoxication using a PDP. The SHAP values for minimum HRs showed significant elevation, with an average increase from approximately 80 bpm to peaks of 90 bpm and reaching up to 100 bpm (ranging from 60 to 120 bpm, with a few data points exceeding 120 bpm). These elevated HRs corresponded to moderate-intensive self-reported marijuana intoxication (SHAP value>0) in young adults compared to other classes (not- and low-intoxicated).

The SHAP values clearly indicate *a* positive increase in minimum HR associated with a higher likelihood of self-reported MI, irrespective of the impact of the sum of minutes spent moving. The total movement time during self-reported MI influenced the rise in minimum HR, as shown in Figure 7 (top left), where the red values represent a maximum of 5 minutes of movement (our analysis uses 5-minute windows). While HR can fluctuate due to various factors, including physical activity, substance use (eg, alcohol), caffeine, meals, and mental state (eg, stress and anxiety), further research is needed to explore these additional influences.

In brief, patterns for the SD of HRs exhibited fluctuations, but, in general, showed an increase when young adults reported MI (Figure 7, top middle). Negative skewness (indicating a “left-skewed” distribution) in HR was consistently associated with MI. This skewness suggests that there were more HR data points on the right side of the mean (indicating that the median was greater than the mean), leading to a distribution stretched toward higher HR values (Figure 7, top right).

#### Decreased Large-Scale Movements

**Figure 8:**
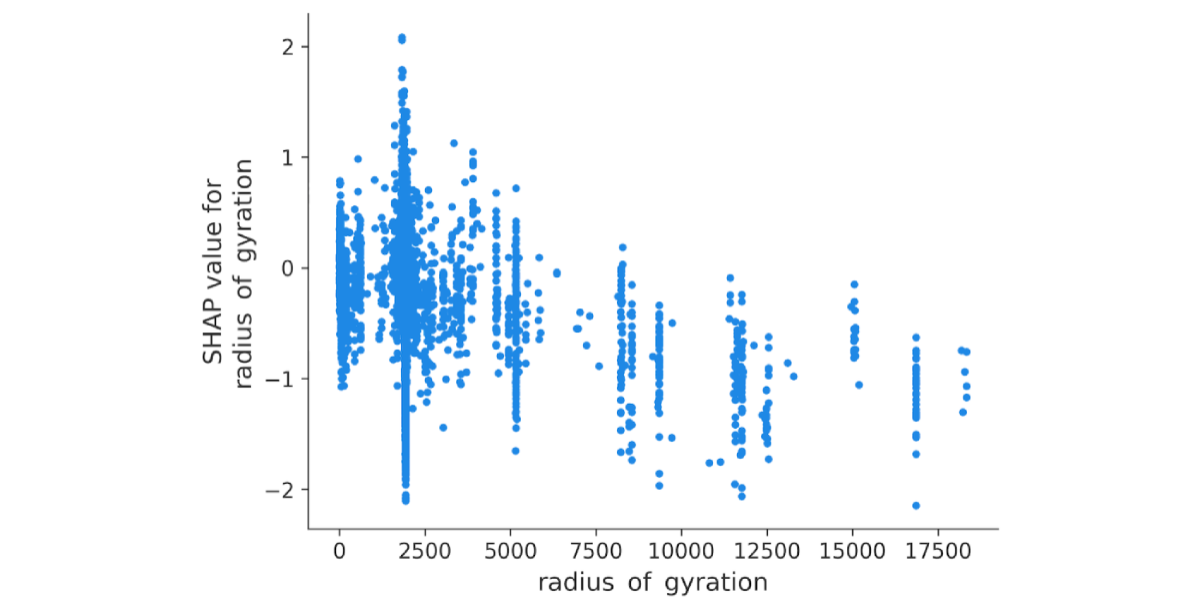
Influence of radius of gyration (unit: meters). SHAP: SHapley Additive exPlanations.

During MI, individuals showed a tendency for limited large-scale movement, often restricted to a radius of approximately 5 km. Notably, instances where the radius of gyration exceeded approximately 10 km were not associated with MI. This finding suggests that when young adults reported MI (rated 4-10), they were less inclined to engage in extensive travel (Figure 8). However, they still demonstrated movement within an average radius of 5 km.

#### Elevated Surrounding Noise Energy

**Figure 9:**
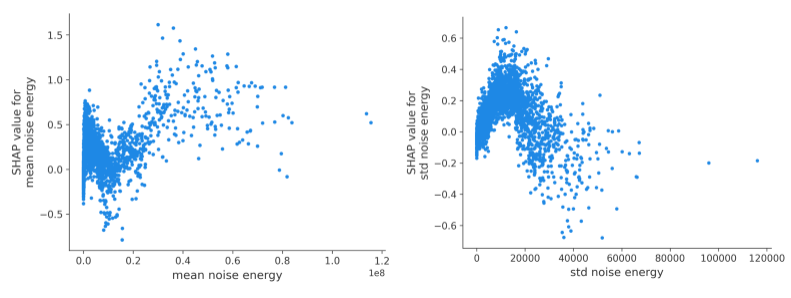
Influence of mean (left) and SD (right) noise energy (unit: Joule). SHAP: SHapley Additive exPlanations.

Interestingly, while the variance in environmental noise energy increased (with data points deviating further from the mean), the average noise energy decreased, though it exhibited an overall upward trend (Figure 9, left). Instances of MI were associated with increased noise variability (calculated based on the amplitude of audio samples), followed by a subsequent reduction (Figure 9, right).

Analyzing ambient sounds provides insights into the environmental context where individuals reporting MI might be located. This could include situations such as marijuana smoking, socializing with friends, or engaging with media like television or music. Although GPS-generated features were the primary indicators, MI may or may not be directly linked to specific locations such as shared social spaces (eg, lounges) or entertaining venues (eg, bars, pubs, or clubs). Nevertheless, it remains plausible that young adults reporting MI may choose to stay in noisy environments.

#### Prolonged Sleep Patterns

**Figure 10:**
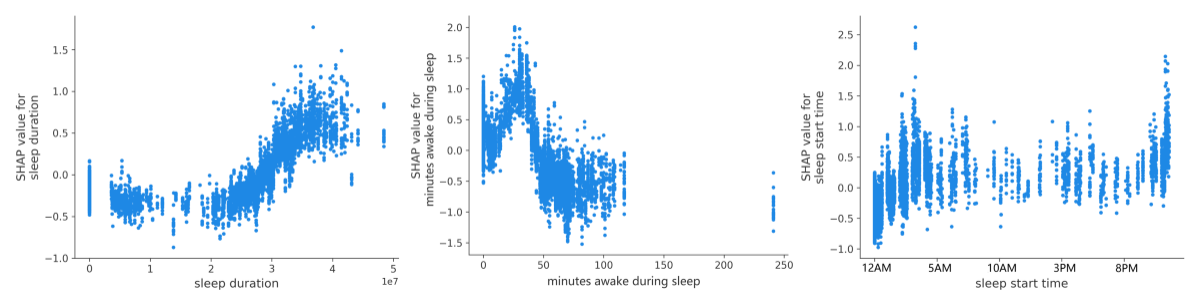
Total sleep duration (left), minutes awake during sleep (middle), and sleep start time (right). SHAP: SHapley Additive exPlanations.

Distinct sleep patterns were linked to episodes of self-reported MI. Individuals who reported MI demonstrated extended sleep durations, spanning approximately 8 to 11 hours (Figure 10, left) the day before self-reported intoxication. In contrast, instances with low or no reported intoxication generally corresponded to healthy sleep durations, averaging around 6-7 hours, with some patterns as short as 2 hours.

There was also a positive correlation between the duration of minutes awake after falling asleep and self-reported MI, particularly when the period involved less than 50 minutes of wakefulness. However, an increase in extended minutes awake after falling asleep (if >50 minutes, extending beyond approximately an hour) did not show any significant association with a likelihood of MI (Figure 10, middle). Regarding sleep start times, the data indicated peaks at both 11 PM and early morning hours, with a rise in sleep start times continuing until around 4 AM (Figure 10, right).

In summary, elevated minimum HR values were clearly linked to a higher likelihood of self-reported MI. However, we observed that GPS-travel patterns (macromovements) did not appear to increase during self-reported marijuana intoxication. Interestingly, extended sleep hours and minutes awake during sleep [40] the day before self-reported marijuana intoxication were associated with MI.

### Additional Analyses for Real-World Feasibility

To enhance the practicality of our ML model in real-world settings, we conducted supplementary analyses to evaluate our top-performing model, the XGBoost-MobiFit model, under different scenarios. These scenarios involved: (1) excluding GPS-derived travel data due to potential privacy concerns or GPS deactivation; (2) excluding sleep data in cases where users did not provide sleep information; and (3) excluding both GPS-derived travel and sleep data. This approach aims to explore the feasibility of offering more flexible data collection options, potentially addressing privacy concerns and incomplete data issues.

In brief, excluding GPS-derived features (XGBoost-MobiFit-GPS excluded) resulted in a 15% decrease in the *F*_1_-score compared to the best model, with a 10% reduction in sensitivity (recall). Excluding sleep data (XGBoost-MobiFit-Sleep excluded) led to a 24% decrease in the *F*_1_-score compared to the best model. When both GPS and sleep features were excluded (XGBoost-MobiFit-GPS-Sleep excluded), the model experienced a 16% reduction in *F*_1_-score and showed the lowest recall for identifying self-reported MI classes compared to the best-performing model. Please refer to Multimedia Appendix 5 for a detailed description of the additional analyses and results.

## Discussion

### Overview

The ability to detect subjective reports of acute marijuana intoxication in natural environments using mobile sensors has the potential to enable just-in-time interventions [41] to reduce marijuana-related harms. To the best of our knowledge, this is the first study that demonstrates the impact of integrating smartphone-based and wearable sensor features on the enhancement of the performance and interpretability of algorithms in detecting acute marijuana intoxication in naturalistic environments.

As hypothesized, we found that the XGB-MobiFit model, which combined smartphone sensor data with Fitbit features outperformed models that used only mobile or only Fitbit data. By integrating sensors from both smartphones and wearable devices, our best-performing algorithm balances specificity and sensitivity on unseen samples, enabling interpretable, transparent, and unobtrusive detection of acute subjective marijuana intoxication in natural environments. This opens up opportunities for real-time monitoring in everyday settings and the implementation of just-in-time adaptive interventions.

XAI visualizations supported our second hypothesis, highlighting HR, GPS, and physical movement data as key features that contributed to self-reported marijuana intoxication predictions. These findings were observed beyond the influences of simply applying time of day and day of the week features (ranked 1st and 4th, respectively), as validated in [11], particularly during instances of self-reported subjective marijuana intoxication in naturalistic environments.

### Interpretable Behavioral and Physiological Signals of Marijuana Intoxication in Real-World Settings

To explain the results of the black-box ML models to detect marijuana intoxication in everyday settings, our study integrated sensors from smartphones and a wearable device, identified key sensor features, and used XAI to facilitate the interpretation of model results. The findings are consistent with prior research conducted in controlled laboratory settings, which consistently found an acute increase in resting HR following marijuana use [12-14]. Our results suggest the potential for HR with behavioral factors to detect marijuana intoxication “outside of laboratory settings” using off-the-shelf devices in naturalistic environments. While many factors can affect HR in daily life, this study yielded significant HR features and insights from the elevated HR patterns during self-reported acute marijuana intoxication. Future research could explore associations between HR and other physiological and behavioral indicators of marijuana use, such as respiration, to better capture marijuana intoxication in natural environments [42]*.*

The use of XAI visualization could help increase transparency and accountability when conducted as part of a substance use detection system [43, 44]. It is promising to use XAI as it enables researchers and clinicians to understand how algorithms arrive at decisions and identify key behavioral and physiological attributes, providing opportunities to improve detection accuracy and enhance trust in the algorithm over time.

### Real-Time Detection and Intervention Potential

Compared to an average 30-minute marijuana episode, the 5-minute window used in the best-performing model is small enough to predict marijuana intoxication in near real-time. Detecting marijuana intoxication in near real-time promotes just-in-time intervention, which serves as a crucial first step toward reducing possible marijuana-related harm in a timely manner.

Our best detection model is unlikely to misclassify a “high” state as not high, which demonstrates the potential for using our detection algorithm with unseen data in real-world contexts. On the unseen test set, we obtained 85% precision (92% precision for 3 classes) in specifically identifying self-reported moderate-intensive marijuana intoxication. Passive sensing using smartphone-based sensors has been investigated in the context of alcohol intoxication [25,26,43], and here we extend this research to self-reported marijuana intoxication [11] beyond smartphone-based sensors, which could ultimately be useful for JIT interventions [41] to reduce marijuana-related harm. The value to society and individuals of reducing marijuana-related harm is clear. If individuals choose to use such a personal detection system, they will need to keep their phone charged and with them when using marijuana and wear a device (eg, Fitbit) and keep it charged as well.

For real-time modeling using the XGBoost algorithm, deploying the estimated model onto a computing device is an indispensable phase. We envisage two primary deployment scenarios: first, local assessments can be generated by deploying the model directly onto users’ devices, such as smartphones. This approach ensures seamless functionality even without an internet connection but requires adequate storage and computational capacity. Second, cloud-based computation can be used. While this approach relies on a stable internet connection, it effectively offloads the computational burden from the user’s device. Real-world applications introduce pragmatic considerations such as battery longevity, which could be affected by the model’s continuous operation, and user privacy during data transmission and generation of model results.

Therefore, a comprehensive assessment of the model’s feasibility in real-time operational settings is important. Our proposed generalized model, designed to operate across a diverse demographic spectrum rather than relying on individual-specific (idiographic) models, offers advantages in terms of scalability and practicality.

### Privacy Considerations and User-Centric Configuration Choices

To highlight the benefits of combining sensor features from both smartphone and wearable devices while addressing potential privacy concerns, particularly related to location data, we aim to offer participants additional configuration choices rather than study withdrawal. For example, participants can deactivate GPS sensors if desired. This is demonstrated by our testing of the best-performing model, XGBoost-MobiFit, where we excluded location features. The analysis revealed a 15% (XGBoost-MobiFit-GPS excluded) decrease in *F*_1_-score from the best model. As proposed by Bae et al [43], collecting GPS data and using rounded GPS data extraction (ie, less precise location data) could be a viable approach. This avoids using raw latitude and longitude, which may contain sensitive information on specific locations. Researchers and clinicians could consider providing alternative options instead of completely disabling GPS, as GPS data contributes to the model’s accuracy.

Moreover, to assess the efficacy of our top-performing model, we conducted tests after excluding sleep-related features (Multimedia Appendix 5). The analysis revealed a 24% (XGBoost-MobiFit-Sleep excluded) decrease in the *F*_1_-score compared to the best model’s performance. While participants may benefit from the option to disable sensors when necessary, it is important to note that this could potentially decrease the model’s ability to detect marijuana intoxication.

By building a system that prioritizes privacy and user autonomy, we can provide a valuable tool to reduce marijuana-related harm to individuals and society. Ultimately, each person will have to decide for themselves whether the benefits of a detection and intervention system outweigh the tradeoffs in minimizing possible marijuana-related harms to themselves and the broader community.

### Limitations and Future Work

The first limitation of this study is relying on self-reporting as the ground truth, which may be subjective. This study extends prior ESM work, which codes self-reported marijuana use as yes or no [45], by asking participants to rate marijuana intoxication from 0 to 10, which may be subject to recall or other biases in reporting. The broad categorization might overlook nuanced differences within three categories: low-intoxication (1-3), moderate-intensive marijuana intoxication (4-10), and not-high (0), which could affect the accuracy of the classifiers. Future analyses examining the performance of mobile and wearable sensors against different thresholds for a subjective marijuana intoxication outcome could be valuable.

Another limitation was the size, diversity, and duration of the participants in the study. Since the participants were all young adults, the finding may not be generalizable to a broader age group. In addition, the level of compliance (63%) in completing the morning, afternoon, and evening surveys is relatively low. Thus, it is unclear whether all episodes of marijuana use were reported by participants, which could limit model performance. However, since there is no real-time accessible biological testing method at the time of publication, validating self-reported data with the current method still represents the best alternative. The current findings warrant future replication in a larger and more diverse group of participants over a longer period to address the limitations and validate the findings.

In addition, our model performed best when tested on the same participants it was trained on (with no overlap between training and testing data). While this has a valid use case, it assumes that we can always collect labeled training data for participants for whom we would like to apply the model. By applying more testing data, using more sophisticated sensor features, and better model tuning, future models could improve generalization over unseen testing participants. The HR data only holds significance when examined together with activity data. An acute increase in HR by itself is nonspecific and may not be associated with marijuana use or intoxication. False alarms triggered by the algorithm could erode trust in an automated system, whereas low sensitivity to actual marijuana use could result in marijuana-related harm. Therefore, it is important to investigate the interplay between human activities associated with marijuana intoxication and physiological signals in a larger population, and how these interactions can contribute to intervention delivery in real-world contexts.

Finally, it is crucial to acknowledge that the potential impact of polysubstance use on the interpretation of physiological signals associated with self-reported cannabis intoxication was not included. While ESM is used to collect information on the use of other substances, our analysis did not account for the effects of polysubstance use due to the limited scope of the study. The presence of polysubstance use could potentially confound the physiological signals attributed to marijuana. This may lead to inaccuracies in our algorithm, particularly in distinguishing between marijuana intoxication and the effects of other substances. Thus, while our study provides valuable insights into self-reported marijuana intoxication, it has limitations in addressing the full spectrum of real-world polysubstance use. Future research should include developing algorithms that can differentiate between the physiological signals associated with different substances, including polysubstance use.

### Conclusions

Our study demonstrates that integrating features from smartphone-based sensors and wearable devices significantly improves the detection of self-reported marijuana intoxication in natural environments among young adults. The XGBoost-MobiFit model, which combines data from both smartphone sensors and wearable devices, achieved an *F*_1_-score of 0.85 in detecting moderate to intensive self-reported marijuana intoxication, outperforming models that relied solely on smartphone sensors. The results suggest that incorporating wearable device data enhances the XGBoost model’s performance by 13%, justifying the additional complexity of using wearable devices among young adults.

Key features contributing to the detection of self-reported “MI” included an acute increase in HR (measured by Fitbit), macromovement indicators (derived from GPS data), and prolonged sleep patterns the night before self-reported marijuana intoxication (measured by Fitbit).

Future research should focus on refining the algorithms that integrate smartphone and Fitbit sensor data in larger, more diverse samples. In addition, exploring how these algorithms, informed by XAI, can support the development of just-in-time interventions for clinicians is essential. Such interventions could offer context-adaptive, personalized strategies to minimize potential marijuana-related harms, such as intoxicated driving, therefore reducing the frequency and severity of acute marijuana-related incidents among young adults.

## Appendix

Multimedia Appendix 1 Extracted features overview.

Multimedia Appendix 2 Feature selection.

Multimedia Appendix 3 Rationale for machine learning model selection.

Multimedia Appendix 4 Comparison of models with different classifiers.

Multimedia Appendix 5 Privacy-preserving XGBoost-MobiFit models.

## Abbreviations

AUC: area under the curve
bpm: beats per minute
ESM: experience sampling method
HR: heart rate
MI: moderate-intensive intoxication
ML: machine learning
PDP: partial dependence plot
SHAP: SHapley Additive exPlanations
THC: delta-9 tetrahydrocannabinol
XAI: explainable artificial intelligence
XGBoost: eXtreme Gradient Boosting
