# Supplementary material for: Enhancing Interpretable, Transparent, and Unobtrusive Detection of Acute Marijuana Intoxication in Natural Environments: Harnessing Smart Devices and Explainable AI to Empower Just-In-Time Adaptive Interventions: Longitudinal Observational Study
Source: JMIR AI. 2025 Jan 2;4:e52270. doi: 10.2196/52270 (PMC11739728; doi:10.2196/52270)
Supplement: Multimedia Appendix 1 [file ai_v4i1e52270_app1.docx]

In our study, data on location, accelerometer, Wi-Fi, and battery usage were collected and analyzed over a five-minute time period using various smartphone sensors. The latitude, longitude, total distance, average speed, movement time, number of position changes, and movement status of the location; the XY, YZ, and ZX angles of the accelerometer as well as the average accelerometer value; the frequency of the Wi-Fi; and the charging and discharging of the battery were collected. These data were statistically analyzed for maximum, minimum, mean, median, and standard deviation. Please refer to Table 1 for additional details.

We collected data on several metrics such as heart rate, steps, resting behavior, distance traveled, and sleep quality of participants over a five-minute time period using a Fitbit device. Heart rate data included maximum heart rate, minimum heart rate, average heart rate, median heart rate, standard deviation of heart rate data, kurtosis, and skewness. Additionally, the resting heart rate of the participants was recorded. Step data included various statistics of step counts, while step speed and walking speed were extracted. Distance traveled was analyzed, including its maximum, minimum, mean, median, and standard deviation. In addition, we calculated the duration, onset, and end times of sleep. Please refer to Table 2 for additional details.

The Fitbit resting HR feature was calculated as follows: after aggregation within a 5-minute window, if a person did not move (ie, no steps) within that window, then the resting HR was computed for that period. The features of ‘pace,’ ‘walk speed,’ and ‘sedentary’ were extracted as follows:

1. Pace: time (min)/distance (meter).
2. Walk speed: 1/pace (meters/min).
3. Moving: At each minute, we checked whether the person was moving. If the number of steps per minute was greater than 0, then that minute was marked as moving (ie, 1); otherwise, it was marked as not moving (ie, 0).
4. Sedentary: For the sedentary feature [58], each minute was checked for a sedentary bout. If the number of steps per minute was 0, then that minute was marked as a sedentary bout (ie, 1); otherwise, it was marked as not a sedentary bout (ie, 0).

Additionally, we extracted the number of minutes participants were awake during the night when their sleep was disrupted the night before a self-reported instance of marijuana intoxication.

**Table S1. Mobile Feature Description**

| **Category** | **Feature** | **Description** |
| --- | --- | --- |
| Location | latitude | The latitude data of the phone was collected, and its maximum, minimum, mean, median, and standard deviation were calculated in five-minute segments [51]. |
| Location | longitude | The longitude data of the phone was collected, and its maximum, minimum, mean, median, and standard deviation were calculated in five-minute segments [51]. |
| Location | total distance | The total distance was calculated using latitude and longitude data, and its maximum, minimum, mean, median, and standard deviation were determined in five-minute segments [51]. |
| Location | speed mean sec | The velocity was calculated using latitude and longitude data, and its maximum, minimum, mean, median, and standard deviation were determined in five-minute segments [51]. |
| Location | moving time | The moving time was calculated based on latitude and longitude data [51]. |
| Location | number location  (ie. number of location changes) | The number of transitions between stationary and moving states (and vice versa) was calculated in five-minute segments. Speeds less than 0.3 m/s were classified as stationary, and speeds greater than 0.3 m/s were classified as moving [51]. |
| Location | moving state | Motion state was classified as 0 if the moving distance was 0, and 1 if the moving distance was greater than 0 [51]. |
| Accelerometer | angle xy | The distance the phone moves in the x-axis and y-axis directions was recorded as a point. A line was drawn from this point to the origin, and the angle between the line and the x-axis was calculated. Its maximum, minimum, mean, median, and standard deviation were calculated in five-minute segments [52]. |
| Accelerometer | angle yz | The distance the phone moves in the y-axis and z-axis directions was recorded as a point. A line was drawn from this point to the origin, and the angle between the line and the y-axis was calculated. Its maximum, minimum, mean, median, and standard deviation were calculated in five-minute segments [52]. |
| Accelerometer | angle zx | The distance the phone moves in the z-axis and x-axis directions was recorded as a point. A line was drawn from this point to the origin, and the angle between the line and the z-axis was calculated. Its maximum, minimum, mean, median, and standard deviation were calculated in five-minute segments [52]. |
| Accelerometer | accelerometer avg | The excitation of the accelerometer on the x-axis, y-axis, and z-axis was measured. The maximum, minimum, mean, median, and standard deviation of these values were calculated in five-minute segments [2]. |
| Wi-Fi | Wi-Fi | The frequency of Wi-Fi connections was counted, and the maximum, minimum, mean, and standard deviation of Wi-Fi frequency were calculated in five-minute segments [2]. |
| Battery | battery charge | The maximum, minimum, mean, and standard deviation of the battery charging time and charge variation were calculated over five-minute durations [2]. |
| Battery | battery discharge | The maximum, minimum, mean, and standard deviation of the battery discharge time and charge variation were calculated over five-minute durations [2]. |

**Table S2. Fitbit Feature Description**

| **Category** | **Feature** | **Description** |
| --- | --- | --- |
| Fitbit heart rate | heart rate | During a five-minute period, we measured the heart rate of the participants and calculated various statistical measures, including the maximum and minimum heart rates, the average (mean) heart rate, the median heart rate, the degree of variation (standard deviation), the 25th percentile (first quartile), the 75th percentile (third quartile), the degree of peakedness (kurtosis), and the degree of asymmetry (skewness) of the heart rate data. In addition, we recorded the resting heart rates of the participants. |
|  | resting heart rate | Participants’ activity status was assessed using their pace data. If no movement was detected for a continuous five-minute period, participants were categorized as being at rest, and their resting heart rate was calculated by averaging their heart rate data over that interval. |
| Fitbit step | steps | The maximum, minimum, mean, median, standard deviation, first quartile, and third quartile of step rates were calculated in five-minute segments. The features ‘pace,’ ‘walk speed,’ and ‘sedentary’ were extracted as follows:   - Pace: time (minutes)/distance (meters). - Walk speed: 1/pace (meters/minute). |
| Fitbit sedentary | sedentary behavior | For the sedentary feature, each minute was checked for a sedentary bout. If the number of steps per minute was 0, that particular minute was marked as a sedentary bout (ie, 1); otherwise, it was marked as not sedentary (ie, 0). The maximum, minimum, mean, median, standard deviation, first quartile, and third quartile of sedentary bouts, breaks, and duration time were calculated for five-minute intervals and over a 24-hour period. |
| Fitbit distance | distance | The maximum, minimum, mean, median, standard deviation, first quartile, third quartile, kurtosis, and skewness of moving distance were calculated in five-minute segments. For ‘moving,’ each minute was checked to determine whether the participant was moving or not. If the number of steps per minute was greater than 0, that particular minute was marked as moving (ie, 1); otherwise, it was marked as not moving (ie, 0). |
| Fitbit sleep | sleep | We calculated the duration of sleep, the start and end times of sleep, and sleep quality. We also extracted the number of minutes participants were awake during the night (time awake after sleep onset, WASO) when their sleep was disrupted the night before the event. |
| Fitbit step | pace and walk | The sum, maximum, minimum, mean, median, standard deviation, first quartile, and third quartile of pace and walk metrics were calculated in five-minute segments. |
