# Supplementary material for: Enhancing Interpretable, Transparent, and Unobtrusive Detection of Acute Marijuana Intoxication in Natural Environments: Harnessing Smart Devices and Explainable AI to Empower Just-In-Time Adaptive Interventions: Longitudinal Observational Study
Source: JMIR AI. 2025 Jan 2;4:e52270. doi: 10.2196/52270 (PMC11739728; doi:10.2196/52270)
Supplement: Multimedia Appendix 2 [file ai_v4i1e52270_app2.docx]

For feature selection using XGBoost, we filtered features with importance greater than 0.005 based on the Gini coefficient. This criterion ensures that only the most influential features are included in subsequent analyses, thereby improving the model's interpretability and efficiency. The screening process carefully evaluated the contribution of each feature to the predictive power of the model and ensured that all retained features significantly impacted the model. After screening, a detailed list was created for each dataset, including the name, classification, and Gini coefficient of each feature. The table below presents the full list, highlighting the statistical significance of each feature and providing a foundation for further analysis and model tuning. The dataset initially included a large number of features: 270 features in total. To enhance accuracy and efficiency, a data cleaning process was conducted to remove highly correlated features. Feature selection was then applied using Gini importance [57] to identify the most relevant features for the analysis. A higher Gini score indicates greater feature importance in classification tasks. After this process, 62 features were selected for the combined dataset (Fitbit and mobile data). Similar optimization processes were applied to subsets of the dataset containing only Fitbit or mobile data.

- Fitbit Dataset: Initially included 90 features; 26 features were retained after cleaning and selection.
- Mobile Dataset: Initially included 182 features; 49 features were retained after cleaning and selection.

This reduction suggests that the optimization process successfully identified a core set of relevant features for each dataset, enabling a more focused and effective analysis. Despite the differences in feature sets, the selected features represent the most relevant variables for the analysis.

1. For the *Mobile* dataset, using machine learning for feature selection, we identified 49 features ranked by their Gini scores, with the highest scores indicating greater importance in classification tasks. The top features included the number of Bluetooth samples (0.0671), walking (0.0600), and the radius of gyration (0.0490). These features span multiple categories, including activity, calls, Wi-Fi, location, and time.
2. For the *Fitbit* dataset, we identified 26 features through machine learning feature selection, ranked by their Gini scores. The top features included sleep start time (0.0957), sleep duration (0.0901), minutes awake during sleep (0.0850), and sleep end time (0.0843). These features span multiple categories, including steps, time, and heart rate. The Fitbit results demonstrated a diverse set of features across these categories, highlighting their significance in predictive modeling.
3. For the *MobiFit* dataset, we identified 64 features using machine learning feature selection. The most important features based on Gini scores included the number of Bluetooth samples (0.0474), walking (0.0458), sleep end time (0.0427), and the number of unique Wi-Fi hotspots (0.0412). These features span multiple categories, including activity, Wi-Fi, steps, keypress, location, time, and heart rate.

The optimized datasets with reduced features are expected to yield more accurate and meaningful results, facilitating better conclusions and informed decision-making. These features encompass a wide range of measurements related to physical activity, communication behaviors, battery usage, and sleep patterns, emphasizing their utility in predictive modeling.


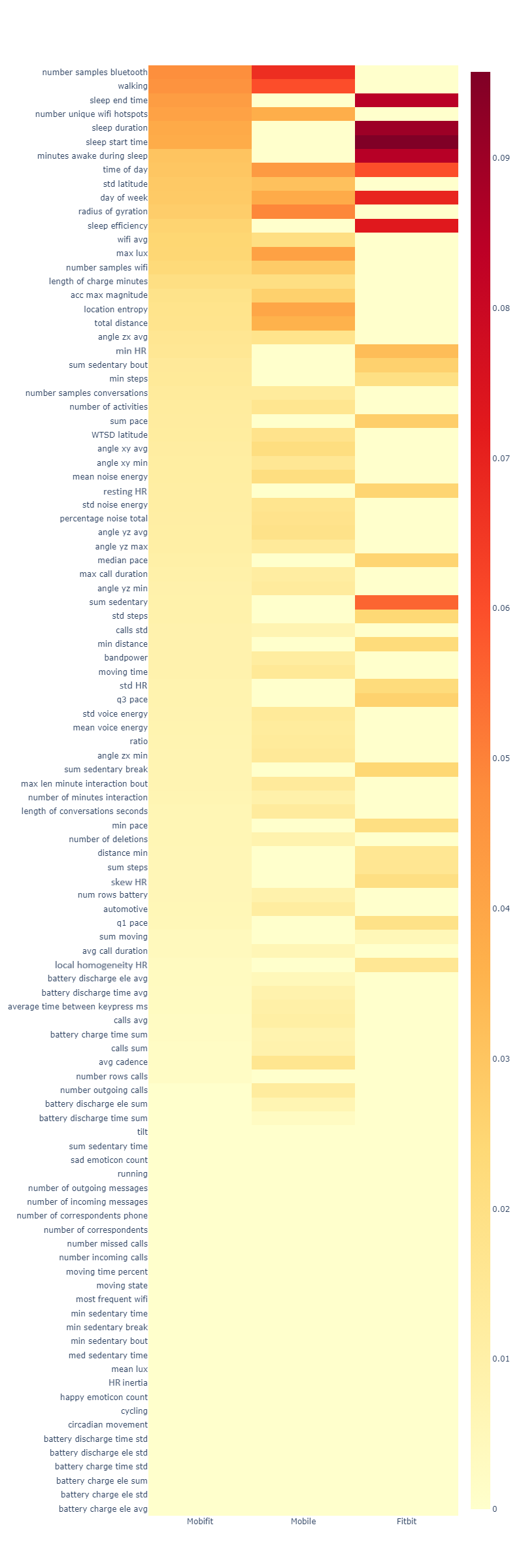


**Figure S1. Selected Features for Modeling**

**Table S1. Mobile Feature Importance**

| **No.** | **Category** | **Feature name** | **GINI score** |
| --- | --- | --- | --- |
| 1 | bluetooth | number samples bluetooth | 0.0671 |
| 2 | activity | walking | 0.0600 |
| 3 | location | radius of gyration | 0.0490 |
| 4 | time | time of day | 0.0435 |
| 5 | light | max lux | 0.0415 |
| 6 | location | location entropy | 0.0399 |
| 7 | time | day of week | 0.0382 |
| 8 | Wi-Fi | number unique Wi-Fi hotspots | 0.0372 |
| 9 | location | total distance | 0.0359 |
| 10 | location | std latitude | 0.0312 |
| 11 | Wi-Fi | number samples Wi-Fi | 0.0281 |
| 12 | accelerometer | acc max magnitude | 0.0264 |
| 13 | accelerometer | angle xy avg | 0.0212 |
| 14 | conversation plug-in | mean noise energy | 0.0210 |
| 15 | battery | length of charge minutes | 0.0204 |
| 16 | Wi-Fi | wifi avg | 0.0203 |
| 17 | accelerometer | angle yz avg | 0.0184 |
| 18 | location | WTSD latitude | 0.0178 |
| 19 | conversation plug-in | percentage noise total | 0.0177 |
| 20 | accelerometer | angle zx avg | 0.0176 |
| 21 | conversation plug-in | std noise energy | 0.0169 |
| 22 | gait | avg cadence | 0.0164 |
| 23 | activity | number of activities | 0.0164 |
| 24 | accelerometer | angle xy min | 0.0154 |
| 25 | location | moving time | 0.0146 |
| 26 | accelerometer | angle zx min | 0.0143 |
| 27 | conversation plug-in | std voice energy | 0.0138 |
| 28 | accelerometer | angle yz max | 0.0138 |
| 29 | conversation plug-in | max len minute interaction bout | 0.0135 |
| 30 | activity | ratio | 0.0134 |
| 31 | conversation plug-in | number samples conversations | 0.0132 |
| 32 | accelerometer | angle yz min | 0.0130 |
| 33 | conversation plug-in | length of conversations seconds | 0.0129 |
| 34 | conversation plug-in | mean voice energy | 0.0128 |
| 35 | call | number outgoing calls | 0.0126 |
| 36 | activity | automotive | 0.0120 |
| 37 | activity | bandpower | 0.0119 |
| 38 | call | max call duration | 0.0117 |
| 39 | call | calls avg | 0.0108 |
| 40 | keypress | average time between keypress ms | 0.0099 |
| 41 | conversation plug-in | number of minutes interaction | 0.0093 |
| 42 | battery | num rows battery | 0.0087 |
| 43 | call | calls sum | 0.0085 |
| 44 | battery | battery discharge time avg | 0.0084 |
| 45 | keypress | number of deletions | 0.0081 |
| 46 | battery | battery charge time sum | 0.0078 |
| 47 | call | calls std | 0.0071 |
| 48 | battery | battery discharge time sum | 0.0068 |
| 49 | call | avg call duration | 0.0059 |

**Table S2. Fitbit Feature Importance**

| **No.** | **Category** | **Feature name** | **GINI score** |
| --- | --- | --- | --- |
| 1 | sleep | sleep start time | 0.0957 |
| 2 | sleep | sleep duration | 0.0901 |
| 3 | sleep | minutes awake during sleep | 0.0850 |
| 4 | sleep | sleep end time | 0.0843 |
| 5 | sleep | sleep efficiency | 0.0729 |
| 6 | time | day of week | 0.0693 |
| 7 | time | time of day | 0.0599 |
| 8 | step | sum sedentary | 0.0554 |
| 9 | heart rate | min HR | 0.0324 |
| 10 | step | sum pace | 0.0273 |
| 11 | step | sum sedentary bout | 0.0263 |
| 12 | step | q3 pace | 0.0261 |
| 13 | step | median pace | 0.0252 |
| 14 | heart rate | resting HR | 0.0252 |
| 15 | step | sum sedentary break | 0.0244 |
| 16 | step | std steps | 0.0241 |
| 17 | step | min distance | 0.0224 |
| 18 | heart rate | std HR | 0.0221 |
| 19 | step | min pace | 0.0205 |
| 20 | heart rate | skew HR | 0.0202 |
| 21 | step | min steps | 0.0198 |
| 22 | step | q1 pace | 0.0188 |
| 23 | step | sum steps | 0.0160 |
| 24 | step | distance min | 0.0158 |
| 25 | heart rate | local homogeneity HR | 0.0155 |
| 26 | step | sum moving | 0.0053 |

**Table S3. MobiFit Feature Importance**

| **No.** | **Category** | **Feature name** | **GINI score** |
| --- | --- | --- | --- |
| 1 | bluetooth | number samples bluetooth | 0.0474 |
| 2 | activity | walking | 0.0458 |
| 3 | sleep | sleep end time | 0.0427 |
| 4 | Wi-Fi | number unique Wi-Fi hotspots | 0.0412 |
| 5 | sleep | sleep duration | 0.0386 |
| 6 | sleep | sleep start time | 0.0379 |
| 7 | sleep | minutes awake during sleep | 0.0303 |
| 8 | time | time of day | 0.0296 |
| 9 | location | std latitude | 0.0288 |
| 10 | time | day of week | 0.0285 |
| 11 | location | radius of gyration | 0.0275 |
| 12 | sleep | sleep efficiency | 0.0253 |
| 13 | Wi-Fi | Wi-Fi avg | 0.0247 |
| 14 | light | max lux | 0.0245 |
| 15 | wifi | number samples Wi-Fi | 0.0231 |
| 16 | battery | length of charge minutes | 0.0201 |
| 17 | accelerometer | acc max magnitude | 0.0182 |
| 18 | location | location entropy | 0.0176 |
| 19 | location | total distance | 0.0173 |
| 20 | accelerometer | angle zx avg | 0.0159 |
| 21 | heart rate | min HR | 0.0155 |
| 22 | step | sum sedentary bout | 0.0141 |
| 23 | step | min steps | 0.0139 |
| 24 | conversation plug-in | number samples conversations | 0.0131 |
| 25 | activity | number of activities | 0.0127 |
| 26 | step | sum pace | 0.0123 |
| 27 | location | WTSD latitude | 0.0122 |
| 28 | accelerometer | angle xy avg | 0.0116 |
| 29 | accelerometer | angle xy min | 0.0115 |
| 30 | conversation plug-in | mean noise energy | 0.0113 |
| 31 | heart rate | resting HR | 0.0112 |
| 32 | conversation plug-in | std noise energy | 0.0112 |
| 33 | conversation plug-in | percentage noise total | 0.0110 |
| 34 | accelerometer | angle yz avg | 0.0109 |
| 35 | accelerometer | angle yz max | 0.0107 |
| 36 | step | median pace | 0.0102 |
| 37 | call | max call duration | 0.0096 |
| 38 | accelerometer | angle yz min | 0.0090 |
| 39 | step | sum sedentary | 0.0089 |
| 40 | step | std steps | 0.0089 |
| 41 | call | calls std | 0.0085 |
| 42 | step | min distance | 0.0085 |
| 43 | activity | bandpower | 0.0085 |
| 44 | location | moving time | 0.0083 |
| 45 | heart rate | std HR | 0.0079 |
| 46 | step | q3 pace | 0.0079 |
| 47 | conversation plug-in | std voice energy | 0.0077 |
| 48 | conversation plug-in | mean voice energy | 0.0076 |
| 49 | activity | ratio | 0.0073 |
| 50 | accelerometer | angle zx min | 0.0072 |
| 51 | step | sum sedentary break | 0.0072 |
| 52 | conversation plug-in | max len minute interaction bout | 0.0071 |
| 53 | conversation plug-in | number of minutes interaction | 0.0069 |
| 54 | conversation plug-in | length of conversations seconds | 0.0063 |
| 55 | step | min pace | 0.0062 |
| 56 | keypress | number of deletions | 0.0062 |
| 57 | step | distance min | 0.0060 |
| 58 | step | sum steps | 0.0058 |
| 59 | heart rate | skew HR | 0.0058 |
| 60 | battery | num rows battery | 0.0057 |
| 61 | activity | automotive | 0.0056 |
| 62 | step | q1 pace | 0.0054 |
