# Supplementary material for: Enhancing Interpretable, Transparent, and Unobtrusive Detection of Acute Marijuana Intoxication in Natural Environments: Harnessing Smart Devices and Explainable AI to Empower Just-In-Time Adaptive Interventions: Longitudinal Observational Study
Source: JMIR AI. 2025 Jan 2;4:e52270. doi: 10.2196/52270 (PMC11739728; doi:10.2196/52270)
Supplement: Multimedia Appendix 3 [file ai_v4i1e52270_app3.docx]

**How similar are the 24 excluded participants to the larger sample?**

The excluded participants were also aged 18–24, with an average age of 20.08 years (SD 1.68). Of these, 18 self-identified as White, 5 as Black, and 1 as another race/ethnicity. On average, this group first used marijuana at the age of 16.75 years (SD 1.53, range=14-20), and their average age of regular marijuana use was 17.21 years (SD 1.35). Within this excluded group, 33% (n=8) reported daily marijuana use, 17% (n=4) reported using it 5–6 times per week, and 50% (n=12) reported using it 2–4 times per week.

**Rationale for selecting machine learning models:**

For our machine learning models, XGBoost was selected for several important reasons:

1. Balancing complexity and performance: XGBoost strikes an effective balance between computational complexity and predictive performance. It can identify non-linear relationships and variable interactions without the high computational demands associated with deep neural networks (DNNs) [72].
2. Improved interpretability and explainability: Compared to DNNs, XGBoost offers greater model transparency, making it easier to interpret and explain its outcomes [73].
3. Reduced risk of overfitting: By incorporating built-in regularization techniques, XGBoost mitigates overfitting, offering a more reliable alternative to complex models like DNNs [74].

XGBoost operates as a distributed gradient boosting framework, optimizing each decision tree to progressively enhance weak learners into robust predictors. It reduces false positives, simplifies label handling, and supports accurate data classification. Furthermore, it integrates seamlessly with cloud-based platforms like AWS, GCE, and Azure [71], which are compatible with real-time data processing tools such as Spark and Flink. With support for multiple programming languages, including Java, Python, R, and C++, XGBoost facilitates cross-platform deployment. Its ability to perform distributed computations for large-scale models is further enhanced by its efficient cache optimization, making it suitable for handling complex datasets [66].
