# Supplementary material for: Enhancing Interpretable, Transparent, and Unobtrusive Detection of Acute Marijuana Intoxication in Natural Environments: Harnessing Smart Devices and Explainable AI to Empower Just-In-Time Adaptive Interventions: Longitudinal Observational Study
Source: JMIR AI. 2025 Jan 2;4:e52270. doi: 10.2196/52270 (PMC11739728; doi:10.2196/52270)
Supplement: Multimedia Appendix 4 [file ai_v4i1e52270_app4.docx]

To determine the most effective model for each machine learning algorithm, we employed identical training and test datasets and conducted ten-fold cross-validation on each training set. Subsequently, the chosen models were applied to make predictions on the test set, yielding the final results. Based on the evaluation metrics and results obtained from the three datasets, the XGB classifier consistently emerged as the superior model for the three classifications (not-intoxicated vs. low-intoxicated vs. moderate-intensive intoxicated).

While the Random Forest classifier and Multilayer Perceptron (MLP) also demonstrated strong performance, the XGB classifier slightly outperformed them in terms of accuracy, personalization, recall, F1-score, and AUC. This indicates its superior ability to correctly classify data and capture complex relationships within the datasets. The strength of the XGB classifier lies in its gradient boosting framework, which effectively manages both bias and variance, resulting in more accurate predictions. Additionally, its ability to handle a variety of data structures makes it a robust and versatile model.

The Light Gradient Boosting Machine (LGBM) classifier also performed well, particularly in terms of precision, recall, F1-score, and AUC. Its consistently strong results indicate its reliability in making accurate predictions. However, the Decision Tree classifier and Gaussian Naive Bayes (NB) models lagged behind the XGB classifier, Random Forest classifier, and LGBM classifier. While these models achieved acceptable accuracy levels, they were less effective in terms of precision, recall, F1-score, and AUC, suggesting a limited capability to capture complex relationships within the data.

Additionally, models using DNN, Lasso, Ridge, ElasticNet, and Logistic Regression underperformed. The F1-score for these models in the MobiFit dataset did not surpass 0.6, with the highest value reaching only 0.57.

In conclusion, considering the performance results, the XGB classifier proved to be the optimal model for the Mobile, Fitbit, and MobiFit datasets among those tested. The XGB classifier exhibited excellent performance across all key evaluation metrics, making it a promising choice for these datasets.

Table S1. Model comparison across different classifiers

| **Model** | **Machine learning** | **Accuracy** | **Precision** | **Recall** | ***F*_1_-score** | **AUC** |
| --- | --- | --- | --- | --- | --- | --- |
| Mobile | Random Forest | 0.97 | 0.91 | 0.41 | 0.47 | 0.88 |
|  | Decision Tree | 0.84 | 0.40 | 0.60 | 0.42 | 0.72 |
|  | LGBM | 0.97 | 0.77 | 0.51 | 0.57 | 0.94 |
|  | GaussianNB | 0.16 | 0.34 | 0.36 | 0.10 | 0.57 |
|  | XGBoost | 0.97 | 0.85 | 0.51 | 0.60 | 0.94 |
|  | MLP | 0.97 | 0.72 | 0.41 | 0.46 | 0.88 |
|  | Lasso | 0.97 | 0.77 | 0.34 | 0.34 | 0.83 |
|  | Ridge | 0.97 | 0.60 | 0.34 | 0.34 | 0.83 |
|  | ElasticNet | 0.97 | 0.43 | 0.34 | 0.34 | 0.83 |
|  | Logistic regression | 0.97 | 0.60 | 0.34 | 0.34 | 0.83 |
| Fitbit | Random Forest | 0.98 | 0.90 | 0.56 | 0.66 | 0.94 |
|  | Decision Tree | 0.95 | 0.55 | 0.70 | 0.60 | 0.77 |
|  | LGBM | 0.98 | 0.92 | 0.56 | 0.66 | 0.97 |
|  | GaussianNB | 0.96 | 0.32 | 0.33 | 0.33 | 0.50 |
|  | XGB | 0.98 | 0.91 | 0.55 | 0.65 | 0.96 |
|  | MLP | 0.97 | 0.53 | 0.37 | 0.39 | 0.88 |
|  | Lasso | 0.97 | 0.32 | 0.33 | 0.33 | 0.68 |
|  | Ridge | 0.97 | 0.32 | 0.33 | 0.33 | 0.69 |
|  | ElasticNet | 0.97 | 0.32 | 0.33 | 0.33 | 0.69 |
|  | Logistic regression | 0.97 | 0.32 | 0.33 | 0.33 | 0.69 |
| MobiFit | Random Forest | 0.97 | 0.90 | 0.52 | 0.61 | 0.96 |
|  | Decision Tree | 0.87 | 0.42 | 0.67 | 0.46 | 0.76 |
|  | LGBM | 0.98 | 0.92 | 0.56 | 0.65 | 0.99 |
|  | GaussianNB | 0.96 | 0.32 | 0.33 | 0.33 | 0.50 |
|  | XGBoost | 0.98 | 0.91 | 0.58 | 0.67 | 0.99 |
|  | MLP | 0.97 | 0.80 | 0.49 | 0.57 | 0.93 |
|  | Lasso | 0.96 | 0.32 | 0.33 | 0.33 | 0.86 |
|  | Ridge | 0.96 | 0.32 | 0.33 | 0.33 | 0.86 |
|  | ElasticNet | 0.96 | 0.32 | 0.33 | 0.33 | 0.86 |
|  | Logistic regression | 0.96 | 0.32 | 0.33 | 0.33 | 0.86 |
