# Supplementary material for: Enhancing Interpretable, Transparent, and Unobtrusive Detection of Acute Marijuana Intoxication in Natural Environments: Harnessing Smart Devices and Explainable AI to Empower Just-In-Time Adaptive Interventions: Longitudinal Observational Study
Source: JMIR AI. 2025 Jan 2;4:e52270. doi: 10.2196/52270 (PMC11739728; doi:10.2196/52270)
Supplement: Multimedia Appendix 5 [file ai_v4i1e52270_app5.docx]

To enhance the real-world applicability of our machine learning model, we conducted an additional analysis to assess our top-performing model, the XGBoost-MobiFit model, under various scenarios: (1) excluding location data, as some individuals might object to GPS collection due to privacy sensitivities or might have disabled it during the study, despite granting consent to researchers; (2) excluding sleep data; and (3) excluding both location and sleep data. This approach aims to explore the feasibility of offering more flexible options for data collection, potentially alleviating privacy concerns.

To clarify the benefits of using two devices' combined sensor features, while eliminating potential privacy concerns related to location and sleep data, this approach may provide more configuration options for participants rather than their dropping out of the study or disabling the GPS sensor. As we tested the best-performing model (i.e., XGBoost-MobiFit) excluding location and sleep features, respectively, the results showed that the performance of the model excluding location features (XGBoost-MobiFit-GPS excluded) decreased by 15% in F1-score, and excluding sleep features (XGBoost-MobiFit-Sleep excluded) decreased by 24% in F1-score, compared to the best model.

By excluding GPS and sleep features, the model (XGBoost-MobiFit-GPS-Sleep excluded) decreased by 16% in F1-score (Table 7), and demonstrated the lowest recall in identifying self-reported moderate-intensive marijuana intoxication classes (Table 8), compared to the best model. In summary, there is a trade-off between the model performance and a privacy-preserving approach. While participants may benefit from such options for disabling sensors when necessary, making them configurable, the predictability of acute marijuana intoxication could decrease.

The findings presented in Table 9 indicate that the exclusion of location features impacted the model’s sensitivity and precision, while sleep-related features played a crucial role in enhancing the model's ability to accurately predict positive instances (recall) from the actual classes. Particularly noteworthy is the shift observed in the false positive rate for predicting instances classified as “moderate-intensive intoxication” – it increased from 13 (Table 3a, all features in the manuscript) to 49 (Table 9, top, features without GPS data) when compared to instances classified as “not-intoxicated.

Table S1. Comparison of privacy-preserving XGBoost^a^-MobiFit models.

| ^b^ML model | AUC^c^ | *F*_1_-score | Recall | Precision | Accuracy |
| --- | --- | --- | --- | --- | --- |
| XGBoost-MobiFit | **0.99** | **0.85** | **0.79** | **0.92** | **0.99** |
| XGBoost-MobiFit-GPS excluded | 0.94 | 0.70 | 0.69 | 0.72 | 0.97 |
| XGBoost-MobiFit-Sleep excluded | 0.91 | 0.61 | 0.66 | 0.57 | 0.95 |
| XGBoost-MobiFit-GPS-Sleep excluded | 0.95 | 0.69 | 0.63 | 0.78 | 0.98 |

^a^XGBoost: eXtreme Gradient Boosting.

^b^ML: machine learning

^c^AUC: Area under the curve

Table S2. Performance comparison of privacy-preserving XGBoost^a^-MobitFit models in detecting the subjective sense of moderate-intensive marijuana intoxication class.

| ML^b^ model | MI^c^ precision | MI recall | MI *F*_1_-score | MI AUC^d^ |
| --- | --- | --- | --- | --- |
| XGBoost^d^-MobiFit | **0.89** | **0.76** | **0.82** | **0.99** |
| XGBoost-MobiFit-GPS excluded | 0.62 | 0.55 | 0.68 | 0.95 |
| XGBoost-MobiFit-Sleep excluded | 0.34 | 0.50 | 0.40 | 0.92 |
| XGBoost-MobiFit-GPS-Sleep excluded | 0.71 | 0.45 | 0.55 | 0.95 |

^a^XGBoost: eXtreme Gradient Boosting.

^b^ML: machine learning

^c^MI: moderate-intensive intoxication.

^d^AUC: Area under the curve

Table S3. Confusion matrix for experiments of privacy-preserving models; XGBoost-MobiFit without GPS data, XGBoost-MobiFit without sleep data, and XGBoost- MobiFit model without GPS and sleep data

|  | | | Predicted | | |
| --- | --- | --- | --- | --- | --- |
|  |  |  | N^a^ | L^b^ | MI^c^ |
|  | | |  |  |  |
| **XGBoost^d^-MobiFit-GPS Excluded** | | | | | |
|  | **Actual** | | | | |
|  |  | N | **6479** | 33 | 49 |
|  |  | L | 38 | **42** | 0 |
|  |  | MI | 63 | 1 | **79** |
| **XGBoost-MobiFit-Sleep excluded** | | | | | |
|  | **Actual** | | | | |
|  |  | N | **6363** | 64 | 134 |
|  |  | L | 35 | **42** | 3 |
|  |  | MI | 70 | 2 | **71** |
| **XGBoost-MobiFit-GPS-Sleep excluded** | | | | | |
|  | **Actual** | | | | |
|  |  | N | **6517** | 19 | 25 |
|  |  | L | 44 | **35** | 1 |
|  |  | MI | 79 | 0 | **64** |

^a^N: not-intoxicated.

^b^L: low-intoxication.

^c^MI: moderate-intensive intoxication.

^d^XGBoost: eXtreme Gradient Boosting.
